# Machine Learning Techniques to Explore Clinical Presentations of COVID-19 Severity and to Test the Association With Unhealthy Opioid Use: Retrospective Cross-sectional Cohort Study

**DOI:** 10.2196/38158

**Published:** 2022-12-08

**Authors:** Hale M Thompson, Brihat Sharma, Dale L Smith, Sameer Bhalla, Ihuoma Erondu, Aniruddha Hazra, Yousaf Ilyas, Paul Pachwicewicz, Neeral K Sheth, Neeraj Chhabra, Niranjan S Karnik, Majid Afshar

**Affiliations:** 1 Section of Community Behavioral Health Department of Psychiatry and Behavioral Sciences Rush University Medical Center Chicago, IL United States; 2 Center for Education, Research, and Advocacy Department of Social and Behavioral Research Howard Brown Health Chicago, IL United States; 3 Department of Internal Medicine Rush University Medical Center Chicago, IL United States; 4 Section of Infectious Diseases and Global Health Department of Medicine University of Chicago Chicago, IL United States; 5 Department of Psychiatry and Behavioral Sciences Rush University Medical Center Chicago, IL United States; 6 Department of Emergency Medicine Rush University Medical College Rush University Medical Center Chicago, IL United States; 7 Division of Pulmonary and Critical Care, Department of Medicine School of Medicine and Public Health University of Wisconsin Madison, WI United States

**Keywords:** unhealthy opioid use, substance misuse, COVID-19, severity of illness, overdose, topic modeling, machine learning, opioid use, pandemic, health outcome, public health, disease severity, electronic health record, COVID-19 outcome, risk factor, patient data

## Abstract

**Background:**

The COVID-19 pandemic has exacerbated health inequities in the United States. People with unhealthy opioid use (UOU) may face disproportionate challenges with COVID-19 precautions, and the pandemic has disrupted access to opioids and UOU treatments. UOU impairs the immunological, cardiovascular, pulmonary, renal, and neurological systems and may increase severity of outcomes for COVID-19.

**Objective:**

We applied machine learning techniques to explore clinical presentations of hospitalized patients with UOU and COVID-19 and to test the association between UOU and COVID-19 disease severity.

**Methods:**

This retrospective, cross-sectional cohort study was conducted based on data from 4110 electronic health record patient encounters at an academic health center in Chicago between January 1, 2020, and December 31, 2020. The inclusion criterion was an unplanned admission of a patient aged ≥18 years; encounters were counted as COVID-19-positive if there was a positive test for COVID-19 or 2 COVID-19 International Classification of Disease, Tenth Revision codes. Using a predefined cutoff with optimal sensitivity and specificity to identify UOU, we ran a machine learning UOU classifier on the data for patients with COVID-19 to estimate the subcohort of patients with UOU. Topic modeling was used to explore and compare the clinical presentations documented for 2 subgroups: encounters with UOU and COVID-19 and those with no UOU and COVID-19. Mixed effects logistic regression accounted for multiple encounters for some patients and tested the association between UOU and COVID-19 outcome severity. Severity was measured with 3 utilization metrics: low-severity unplanned admission, medium-severity unplanned admission and receiving mechanical ventilation, and high-severity unplanned admission with in-hospital death. All models controlled for age, sex, race/ethnicity, insurance status, and BMI.

**Results:**

Topic modeling yielded 10 topics per subgroup and highlighted unique comorbidities associated with UOU and COVID-19 (eg, HIV) and no UOU and COVID-19 (eg, diabetes). In the regression analysis, each incremental increase in the classifier’s predicted probability of UOU was associated with 1.16 higher odds of COVID-19 outcome severity (odds ratio 1.16, 95% CI 1.04-1.29; *P*=.009).

**Conclusions:**

Among patients hospitalized with COVID-19, UOU is an independent risk factor associated with greater outcome severity, including in-hospital death. Social determinants of health and opioid-related overdose are unique comorbidities in the clinical presentation of the UOU patient subgroup. Additional research is needed on the role of COVID-19 therapeutics and inpatient management of acute COVID-19 pneumonia for patients with UOU. Further research is needed to test associations between expanded evidence-based harm reduction strategies for UOU and vaccination rates, hospitalizations, and risks for overdose and death among people with UOU and COVID-19. Machine learning techniques may offer more exhaustive means for cohort discovery and a novel mixed methods approach to population health.

## Introduction

### Background

The COVID-19 pandemic has illuminated health disparities and inequities in the United States [1-3]. Chronic illness and conditions like diabetes, hypertension, cancer, autoimmune disease, and obesity, often disproportionate in aging and in uninsured populations, are associated with more severe COVID-19 outcomes [4-9]. Derived from electronic health record (EHR) data that were deidentified and aggregated on the TriNetX Research Network platform, national cohort studies have established substantial evidence of increased risks for acquiring COVID-19 and having more severe outcomes for patients with diagnosed mental health disorders or substance use disorder (SUD) [10-12]. Patients with SUD and COVID-19 have a higher odds risk for hospitalization, receiving mechanical ventilation, and mortality [11,13]. Fully vaccinated patients with SUD also have a higher odds risk for COVID-19 breakthrough infections compared to patients with no SUD [12].

Patients with opioid use disorder (OUD) often have comorbidities, such as kidney, pulmonary, liver, cardiovascular, metabolic, and immune-related disorders, that lead to disproportionate susceptibility to COVID-19 [10]. Excessive opioid use has been shown to suppress the immune system and damage the lungs, leading to an impaired respiratory system. These comorbidities could explain the observed severity of clinical outcomes in patients with OUD [11]. In one national study, patients with OUD had the greatest odds risk for breakthrough COVID-19 among those with SUD, and this disparity widened when evaluating outcomes across strata of race/ethnicity and gender. African American patients with OUD displayed an increased risk for acquisition and adverse outcomes [10,12]. Prior to the pandemic, people who misuse opioids were already experiencing the highest number of overdose deaths ever reported [1]; the pandemic has since created new and exacerbated existing disruptions in access to treatment of OUD, further accelerating the rise in overdose deaths [14-17]. COVID-19 has stressed the capacities of emergency departments (EDs) and acute care settings to conduct, for example, manual screenings for SUD, widening treatment gaps for OUD [18]. The higher risk for infection and adverse outcomes, in combination with missed treatment opportunities and increasing overdose deaths, further compounds the negative effects of the pandemic in this already vulnerable population.

Patients who misuse opioids and experience other mental health conditions may struggle with social distancing and quarantine requirements. These patients frequently experience socioeconomic and societal disadvantages that result in crowded living spaces, such as encampments, homeless shelters, and incarceration [12,19]. Stigma around opioid misuse and implicit and structural biases of the health care system could also contribute to the severity of COVID-19 clinical outcomes seen in patients with OUD [20]. Mistrust of health care providers can delay treatment-seeking at the onset of symptoms, further exacerbating illness severity [16,21]. In addition, the pandemic has disrupted access to treatments like buprenorphine, as well as access to methadone, a highly regulated medication for OUD (MOUD) that is disproportionately prescribed to Medicaid patients and may be a driver of the increase in overdose deaths [22,23].

### Objective

Our recent study of unhealthy alcohol use (UAU) among our COVID-19 patients guided our current aims and our use of the term “unhealthy opioid use” (UOU) [13]. Similar to opioid misuse, people with UOU may not have an OUD diagnosis; the US Preventive Services Task Force defines UOU as the consumption of illegally obtained opioids or the nonmedical consumption of prescription opioids [24]. To discern any unique clinical presentations of UOU and COVID-19, we conducted topic modeling from the clinical notes of the EHRs of 2 subcohorts of hospitalized patient encounters: (1) UOU and COVID-19 and (2) no UOU and COVID-19. Next, we tested the association between increasing probability of UOU with increased severity of COVID-19–related health outcomes. Our findings from this novel mixed methods approach may offer more effective COVID-19 prevention and treatment pathways, as well as more effective harm reduction resources and treatment planning for UOU.

## Methods

### Setting and Sample

This cross-sectional study took place at Rush University Medical Center (RUMC), a large academic health center on Chicago’s West Side, and was conducted with data from 4110 inpatient EHR encounters between January 1, 2020, and December 31, 2020. The inclusion criteria were an unplanned admission of a patient aged ≥18 years and a COVID-19 diagnosis. Encounters were counted as COVID-19–positive according to the National COVID Cohort Collaborative phenotype; specifically, encounters were positive if there was a documented positive test for COVID-19 or if 2 or more COVID-19–related International Classification of Disease, Tenth Revision (ICD-10) codes were recorded in a single encounter or day [25]. Using a predefined cutoff with optimal sensitivity and specificity to identify UOU, we ran our Substance Misuse and Referral to Treatment Artificial Intelligence (SMART-AI) classifier on all EHR clinical notes for patients with COVID-19 to estimate a subcohort of patients with UOU and a subcohort with no UOU.

### SMART-AI for Cohort Discovery and Natural Language Processing of Clinical Notes

The SMART-AI classifier is a multi-label convolutional neural network model that was developed and tested within RUMC and externally validated at the trauma center of another local academic health system [26]. SMART-AI demonstrated good face validity, with model features containing explicit mentions of opioid misuse, and demonstrated excellent test characteristics in identifying cases of UOU when validated against the Drug Abuse Screening Test [18,26]. During temporal validation, the sensitivity and specificity for opioid misuse were 0.87 (95% CI 0.84-0.90) and 0.99 (95% CI 0.99-0.99), respectively. The positive predictive value and negative predictive value were 0.76 (95% CI 0.72-0.88) and 0.99 (95% CI 0.99-0.99), respectively. The classifier was trained as a single model with binary outputs for alcohol, opioid-drug, and nonopioid-drug misuse and allows for deactivation of any label; in this study, only the opioid label operated for the purpose of subcohort discovery among the cohort of 2020 COVID-19 hospitalized patients, and the nonopioid drug and alcohol labels were deactivated.

Natural language processing of the sample’s clinical notes used the Clinical Text and Knowledge Extraction System (cTAKES) version 4.0 [27]. The cTAKES is a natural language processing system designed for knowledge extraction from the EHR clinical narrative that is scalable, comprehensive, robust, and interoperable. The cTAKES recognizes words and phrases from the clinical narrative that represent domain concepts, or named entities, in the National Library of Medicine Unified Medical Language System metathesaurus of medical ontologies. These domain concepts have been mapped from clinical notes and standardized as concept unique identifiers (CUIs).

### Ethical Considerations

This study was approved by the RUMC Institutional Review Board (18061108-IRB01). Our sample was drawn from retrospective encounters documented in the EHRs; these data were deidentified for both sets of analyses and did not require informed consent.

### Topic Modeling to Identify Subcohort Clinical Presentations

A domain of unsupervised machine learning, topic modeling synthesizes unwieldy textual data into more concise and deliverable concepts and organizes them into domains, or topics, based on the patterned clustering of the concepts across a data set [28,29]. In our experiment, topic modeling mined the corpus of clinical notes in the EHRs for common groupings of terms, represented as standardized medical concepts, or CUIs. When conducted for each of the 2 subcohorts, this process clustered similar and correlated concepts into topic groupings derived from clinical notes during the 2020 pandemic year, delineating key clinical differences and similarities.

We used latent Dirichlet allocation (LDA) to model the corpus of clinical data from each subcohort. Although more recent models and techniques have achieved higher accuracy, LDA is one of the most effective unsupervised probabilistic topic models for text mining based on CUIs. LDA requires a predefined number of topics to model [29], and coherence value (CV) scores for each subcohort were derived in order to identify the number of topics with the best model fit. Ten topics were determined to be optimal and parsimonious (Figure S1 and Table S1 in Multimedia Appendix 1). Similar to a scree plot in factor analysis, the point at which the CV curve initially bends or plateaus for each subcohort is an indicator of the optimal topic number.

A panel of 6 clinical experts, from 3 academic health centers, including RUMC, in psychiatry, infectious disease, addiction medicine, nursing, pulmonology/critical care, and emergency medicine convened to review and summarize the 10 topics that contained clusters of medical concepts generated for each subcohort. Each topic was presented in word cloud format in order to visually highlight the high-frequency concepts that, in aggregate, formed the core idea or topic (for the complete set of 20 word clouds, see Figure S2 in Multimedia Appendix 2) [30]. Together, the group discussed and agreed upon the emergent topic of the 10 clusters of concepts for each of the patient subcohorts. These topics were written up for the panel’s review, feedback, and to confirm consensus.

### Measurement and Statistical Analysis

#### Measurements

To assess descriptive statistics and test associations with COVID-19 outcome severity, demographic and clinical data were extracted from the EHRs. The variables included age, sex, race/ethnicity, insurance status, length of stay in days, minimum oxygen saturation level, and BMI. COVID-19 severity was measured according to the maximum level of care that a patient received: (1) low severity was an unplanned admission without receiving mechanical ventilation; (2) medium severity was an unplanned admission with receiving invasive mechanical ventilation; and (3) high severity was an unplanned admission ending in death.

#### Primary Outcome Analysis

In order to accommodate some repeated observations and the ordered categorical nature of how severity was measured, mixed effects ordinal logistic regression analyses with random intercepts were conducted to predict COVID-19 severity status of the 2 COVID-19 subgroups. In the first analysis, the classifier’s predictive probability of UOU for each encounter with COVID-19 was regressed onto the severity outcome (ie, low, medium, or high). A higher predictive probability from the classifier indicated a greater likelihood of UOU. In the second analysis, the severity outcome was dichotomized into low (unplanned admission only) and high (unplanned admission with ventilator or in-hospital death). The classifier estimation of UOU probability was log transformed due to strong positive skew in the distribution of probabilities. All models controlled for BMI, age, sex, race/ethnicity, and insurance status. Due to sparse data, the model did not control for smoking status. We also examined interactions between classifier status and these demographic characteristics to test for potential effect modification, though we did not identify any significant interactions, and they are not reported here. Among variables used in the analysis, BMI was missing for 601/4110 (14.6%) of the COVID-19 encounters. Because BMI was not missing at random and missingness was associated with higher outcome severity, complete case analysis was used. Analyses were conducted in Stata (version 17, StataCorp LLC).

## Results

Descriptive characteristics of unplanned admissions in 2020 are presented in Table 1, stratified by UOU and COVID-19 (n=102) and no UOU and COVID-19 (n=4008), with *P* values provided for the chi-square and Kruskal-Wallis tests. Compared to the no UOU subgroup, the UOU subgroup was disproportionately younger (mean age 55.6, SD 14.6 years; *P*=.001), male (68/102, 66.7%; *P*=.002), Black (71/102, 69.6%; *P*<.001), and Medicaid-insured (67/102, 65.7%; *P*<.001). This group was also disproportionately discharged against medical advice (14/102, 13.7%, *P*<.001) and had a significantly shorter average length of stay (mean 6.8, SD 7.9 days; *P*<.001). This subgroup’s BMI (mean 26.3, SD 7.0 kg/m^2^; *P*<.001) and minimum level of oxygen saturation (mean 81.6%, SD 11.6%; *P*=.008) were also lower.

**Table 1:** Sample characteristics for a cohort with unplanned admissions at a Chicago academic health center between January 1 and December 31, 2020 (N=4110), comparing those with unhealthy opioid use and COVID-19 and those with no unhealthy opioid use and COVID-19. Test statistic values represent Kruskal-Wallis tests for continuous variables and the proportion of male patients and chi-square tests for other categorical variables.

Characteristics		UOU^a^ (n=102)	No UOU (n=4008)	Test statistic value (*df*)		*P* value	
Age (years), mean (SD)		55.6 (14.6)	59.4 (17.4)	10.7 (1)		.001	
Sex (male), n (%)		68 (66.7)	2036 (50.8)	9.4 (1)		.002	
**Race/ethnicity, n (%)**					39.5 (3)		<.001
	Black	71 (69.6)	1674 (41.7)				
	White	19 (18.6)	816 (20.4)				
	Hispanic or Latinx	5 (4.9)	1187 (29.6)				
	Other	7 (6.8)	331 (8.2)				
**Insurance status, n (%)**					41.7 (3)		<.001
	Medicaid	67 (65.7)	1402 (34.9)				
	Medicare	20 (19.6)	1366 (34.1)				
	Private	13 (12.7)	906 (22.6)				
	Other	2(1.9)	334 (8.3)				
**Discharge status, n (%)**					147.5 (4)		<.001
	Home	42 (41.2)	1810 (45.2)				
	Other	24 (23.5)	1236 (30.8)				
	Long- or short-term care	13 (12.7)	617 (15.4)				
	In-hospital death	9 (8.8)	312 (7.7)				
	Against medical advice	14 (13.7)	33 (<1)				
Length of stay (days), mean (SD)		6.8 (7.9)	8.5 (10.1)	9.9 (1)		.002	
BMI (kg/m^2^), mean (SD)		26.3 (7.0)	32.0 (10.3)	36.1 (1)		<.001	
Minimum oxygen saturation (%), mean (SD)		81.6 (11.6)	83.4 (12.4)	6.9 (1)		.008	

^a^UOU: unhealthy opioid use.

### Topic Modeling

Our panel characterized the 10 topics modeled from each of the 2 EHR patient encounter subcohorts with COVID-19 in 2020 (Table 2).

For the no UOU subcohort, concepts within each topic spanned a range of symptoms, comorbidities, and procedures indicative of moderate to high severity. The first topic was deemed a “classic hospitalized COVID patient” by the expert panel of physicians and advanced practice nurses and displayed several comorbidities and procedures, such as diabetes and intubation, respectively, associated with higher severity. The second topic was related to sepsis, followed by a topic for ordering procedures associated with COVID-19. Topics 4 through 6 were long-term intensive care unit (ICU) patients, chronic obstructive airway disease, and procedures and interventions to address acute respiratory failure and hypoxia, respectively. Topics 7 through 10 were neurology-related, followed by chronic conditions associated with severe COVID-19 (eg, diabetes, coronary artery disease, and heart failure), then COVID-19–related terms indicating less severity (eg, normal limits, c-reactive protein, and myalgia), and finally conditions highly susceptible to COVID-19, like cancer and organ transplantation.

**Table 2:** Topic modeling for 2020 hospital admissions comparing 10 topics for 2 COVID-19 patient encounter subcohorts: those with unhealthy opioid use and those with no unhealthy opioid use (N=4110). Subcohorts were identified using the Substance Misuse and Referral to Treatment Artificial Intelligence (SMART-AI) digital classifier for opioid misuse [26]. The topic numbers are labels and do not reflect a ranking of topics.

Topic			Concepts
**Unhealthy opioid use (n=102)**			
	1	Cardiopulmonary illnesses and social determinants of health	
	2	Opioid misuse comorbidities	
	3	Renal and cardiac pathologies, HIV-related terms	
	4	Neurological comorbidities with altered mental status	
	5	Neurological workups with cardiac disturbances	
	6	Critically ill/intensive care unit patients	
	7	Risk for overdose with cardiopulmonary and respiratory distress	
	8	Chronic opioid misuse with respiratory distress	
	9	Opioid overdose patients	
	10	Chronic illness and traumatic injury	
**No unhealthy opioid use (n=4008)**			
	1	Classic COVID-19 hospitalization with severity	
	2	Sepsis-related, less clearly COVID-19 related	
	3	COVID-19 orders/procedures, moderate to severe neurological orders	
	4	Long-term intensive care unit patients	
	5	Chronic obstructive pulmonary disease	
	6	Interventions for acute respiratory failure	
	7	Very neurologically focused, less COVID-19 related	
	8	Chronic conditions associated with severe COVID-19	
	9	Less severe COVID-19 symptoms and measures	
	10	Chronic disease highly susceptible to COVID-19	

In the UOU subcohort, topics indicated illness associated with both UOU and COVID-19, as well as social determinants of health. The first topic indicated a number of cardiac and pulmonary chronic illnesses that could increase risk for COVID-19 severity, plus methadone. The second topic was characterized as UOU comorbidities and included concepts like cocaine, methadone, suboxone, and anxiety. Topic 3 was renal and cardiac pathologies with some HIV-related concepts, followed by a topic related to neurological workups and altered mental status. Concepts related to fentanyl, cocaine, Narcan, magnetic resonance imaging, and computed tomography scans of the brain had small-to-medium sized weights relative to heavily weighted concepts for cerebrovascular accidents, angiograms, hemorrhage, stenosis, and seizures. Topic 5 was also deemed to be neurological-related but with blood and cardiac disturbances present, plus methadone. Topic 6 was deemed critical illnesses or ICU patients, with concepts like malnutrition, nutrition function, cardiac arrest, and severe or moderate adverse events prominent in the word cloud. The panel characterized topic 7 as overdose risk with cardiopulmonary disorders, and respiratory and reactive airway terms, like asthma and nebulizer, appeared alongside UOU terms, such as opioids and methadone. Topic 8 was characterized as chronic UOU with respiratory distress, while topic 9 indicated opioid overdose with 3 heavily weighted concepts: Narcan, falls, and respiratory failure. The final topic for the UOU patients was much less distinct, with a mix of chronic illness– and traumatic injury–related concepts along with unhealthy substance use–related concepts like naloxone and liver cirrhosis.

### Mixed Effects Ordinal Logistic Regression

**Figure 1:**
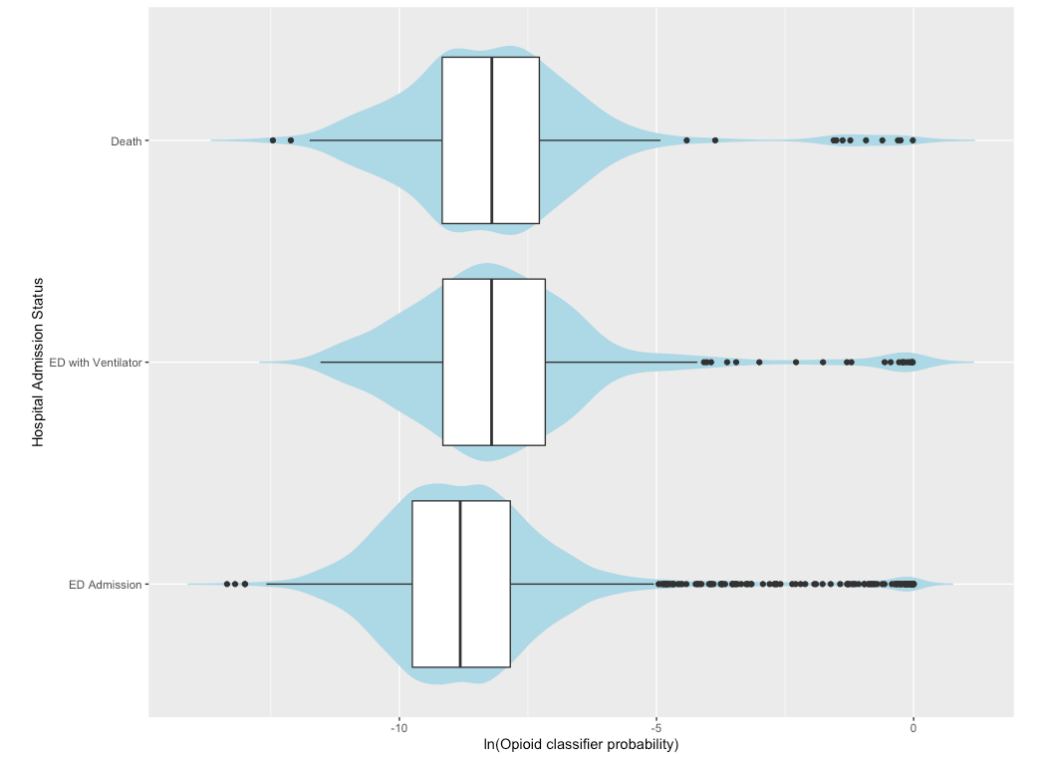
The increased probability of unhealthy opioid use (as a continuous scale) across patient encounters that included a diagnosis of COVID-19 in 2020 (N=4110) was associated with increased outcome severity, measured by unplanned admission via the emergency department at a large Chicago hospital. ED: emergency department. ln: natural log.

**Figure 2:**
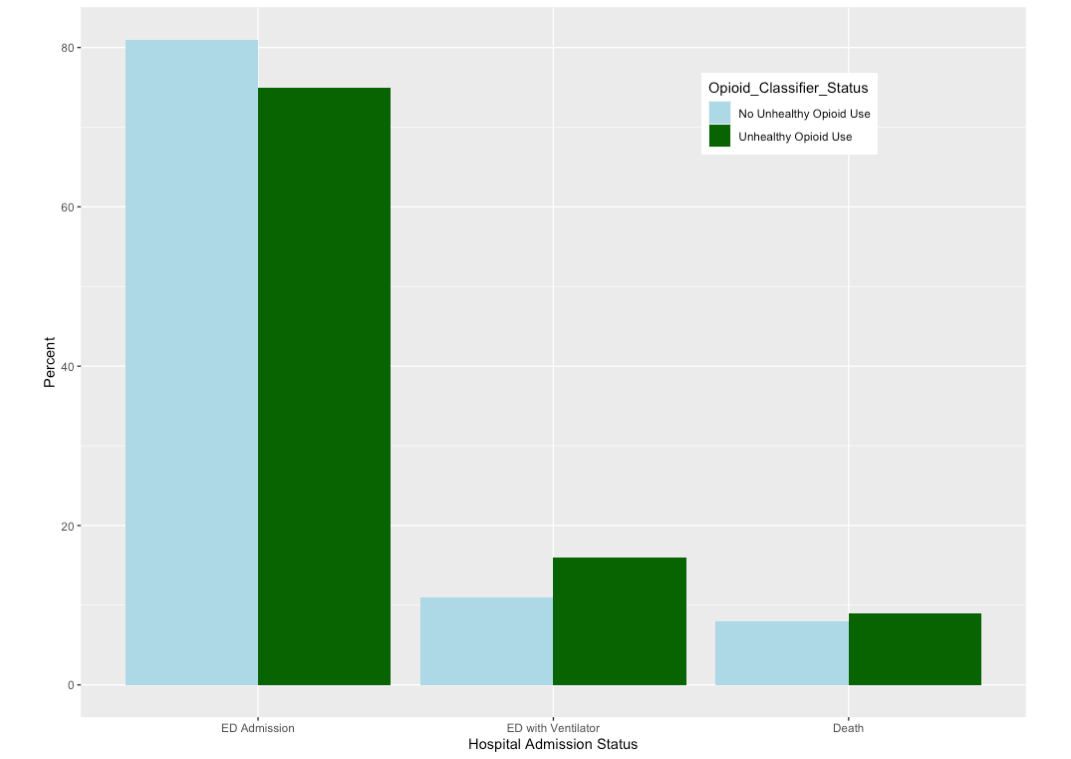
Unplanned hospital admission status via emergency department for patient encounters carrying a diagnosis for COVID-19 (N=4110) at a Chicago hospital in 2020, stratified by unhealthy opioid use and no unhealthy opioid use. ED: emergency department.

In our test for an association of UOU with COVID-19 outcome severity, each incremental increase in SMART-AI’s predicted probability of UOU was associated with higher severity of outcomes (odds ratio [OR] 1.16, 95% CI 1.04-1.29; *P*=.009; Figure 1 and Table 3). Age, sex, and BMI, but not race/ethnicity or insurance status, were also associated with severity status, with male, older, and higher-BMI participants having greater risk of being in more severe categories (Table 3). Results indicating greater severity for COVID-19 patients with UOU were also robust for the dichotomization of severity level into inpatients with no ventilator use or those with either ventilator use or in-hospital death. UOU status remained a predictor of severity in the adjusted analysis (OR 1.19, 95% CI 1.12-1.26; *P*<.001) for the composite dichotomous outcome. The distribution of type of unplanned admission via ED stratified by UOU or no UOU is shown in Figure 2. For admissions with UOU, 77/102 (75%) were ED to hospital admissions, 16/102 (16%) were ED to hospital admissions requiring invasive mechanical ventilation, and 9/102 (9%) were in-hospital deaths. For admissions with no UOU, 3260/4008 (81%) were ED to hospital admissions, 436/4008 (11%) were ED to hospital admissions requiring mechanical ventilation, and 312/4008 (8%) were in-hospital deaths (see Figure 2).

**Table 3:** Adjusted associations between unhealthy opioid use and outcome severity for hospitalized patient encounters carrying a diagnosis of COVID-19 in Chicago between January 1, 2020, and December 31, 2020 (N=4110).

Predictor			Odds ratio (95% CI)		*P* value
Unhealthy opioid use^a^			1.16 (1.04-1.29)		.009
Age			1.01 (1.01-1.02)		<.001
BMI			1.02 (1.01-1.03)		<.001
Sex^b^			0.75 (0.63-0.90)		.002
**Race/ethnicity^b^**					.21
	Black	0.92 (0.73-1.18)		.52	
	Hispanic or Latinx	1.12 (0.84-1.45)		.39	
	Other	1.22 (0.86-1.72)		.27	
**Insurance^b^**					.18
	Medicare	0.87 (0.69-1.07)		.22	
	Private	0.79 (0.62-1.00)		.053	
	Other	0.78 (0.55-1.11)		.17	

^a^Opioid misuse classifications were log transformed in this analysis.

^b^These rows report the *P* value for the omnibus effect for categorical predictors with more than 2 levels, and rows nested with them represent comparisons with the reference categories of male, non-Hispanic White, and Medicaid.

## Discussion

### Key Findings

Our study used SMART-AI, a validated substance misuse classifier, for UOU cohort discovery and to determine whether UOU was an independent predictor of COVID-19 outcome severity. Controlling for age, sex, race/ethnicity, insurance status, and BMI, the regression analysis demonstrated that UOU was an independent risk factor associated with increased severity of COVID-19 outcomes, measured in terms of hospital utilization. This “unhealthy opioid use” category expands the bounds for meeting the threshold for opioid misuse, traditionally a formal OUD diagnosis, and represents a unique contribution to recent studies documenting the association between OUD and COVID-19 outcome severity. As an open-source tool that has high accuracy and no major inequities across demographic subgroups for type I and II errors [26], SMART-AI is a useful and effective tool for both clinical screening and research into substance misuse. This analytic strategy integrating deep learning and unsupervised topic modeling is a novel mixed methods approach.

Our unique application of topic modeling enabled our expert panel to conduct a timely analysis of the 2020 COVID-19 patient data and to distinguish the clinical profile of COVID-19 patients hospitalized with UOU from those with COVID-19 who did not misuse opioids. Across both subgroups of COVID-19 admissions, topics reflected severity but with some distinctly different comorbidities that may have contributed to severity. The UOU subgroup had chronic and acute illnesses related to perivascular, pulmonary, HIV, and psychiatric comorbidities, as well as social determinants of health. The prominence of the Medicaid, methadone, and overdose concepts, for example, indicated a UOU subgroup with high poverty and limited access to health care and other resources who may have experienced medical emergencies due to disruptions in access to opioids or opioid treatments or increased exposure to the community spread of COVID-19 [22,23,31].

The no UOU and COVID-19 subgroup was distinguished by the presence of a sepsis topic and a topic related to less severe COVID-19 symptoms and measures. Consistent with that subgroup’s higher mean BMI and older mean age were the prominence of age-related illnesses, like dementia and sepsis, and weight-related concepts, like diabetes and sleep apnea [32].

### Comparisons With Other Work

Our analysis confirms the presence of a range of chronic illnesses associated with COVID-19 [2,3]. Although race/ethnicity and insurance status were not associated with severity in our analysis, this may be because COVID-19 disproportionately impacts populations on Medicaid or Medicare and Black and Latinx populations at every level of severity in our sample. Nonetheless, the UOU subgroup was disproportionately Medicaid-insured and Black. Further, the prevalence of the topic methadone, versus suboxone, across the UOU subgroup, for example, signals underresourced and underinsured patients who may experience challenges with social distancing and heightened difficulties with access to MOUDs [16]. The distinct presence of both an overdose topic and an overdose risk topic indicates that social determinants of health continue to play a role during the pandemic. The presence of these topics may also indicate disruptions in access to both MOUDs and illicit opioids; both types of disruptions may limit capacity to social distance and increase possible exposure to COVID-19 [14,31].

As with UAU, identified in a companion study conducted by members of our lab, UOU interferes with immune and respiratory functioning and may increase susceptibility to, as well as the severity of, COVID-19 [13]. Taken together, our studies’ methods and findings inform a data-driven approach for timely and effective planning and deployment of resources to improve treatment pathways and outcomes for both unhealthy substance use and COVID-19 [19].

### Limitations

These analyses have limitations. The use of SMART-AI for UOU subcohort discovery could have resulted in the possible misclassification of the cohorts with UOU and no UOU; although SMART-AI has high accuracy, classification also depends on the substance of the documentation in clinical notes.

The 2020 EHR encounter data predate vaccines and new variants of the virus; it is important for future research to index the evolving pandemic, vaccination rates among those with UOU, and changes in UOU and COVID-19 severity. The encounter data were cross-sectional and prevented causal inference of outcome severity. For example, the topic modeling experiment highlighted a distinct topic for opioid overdose and COVID-19. These patients may have been incidentally diagnosed with COVID-19 during hospitalization, complicating the interpretation of outcome severity as associated with COVID-19 rather than with an overdose. UOU also tends to drive higher discharges against medical advice, limiting interpretation of shorter average lengths of stay or low severity outcomes [33,34]. The regression analysis did not adjust for patient comorbidities, and the complete case analysis to address nonignorable missingness of BMI data may have inflated standard errors for the BMI covariate estimate.

Our topic modeling experiment was limited by the number of topics we chose (ie, 10). In addition to CV scores, parsimony and cognitive load for the panelists guided the determination of the optimal topic number. Although bidirectional encoder representations from transformers (BERT) has outperformed cTAKES in terms of distinguishing social and nonsocial sentences and concepts, BERT has a higher computational cost, and cTAKES protects patient privacy with the use of standardized concepts (ie, CUIs) [27,35].

### Conclusion

The role of COVID-19 therapeutics and inpatient management of acute COVID-19 pneumonia remains unclear for patients with UOU [36-38]. The increased risks for severe outcomes, such as invasive ventilation and death, for patients with both COVID-19 and UOU warrants additional considerations for clinical practice and research priorities. Further research is needed to test associations between expanded evidence-based harm reduction strategies for treatment of UOU, vaccination rates, hospitalizations, and risks for overdose and death among people with UOU and COVID-19 [22,23,39,40]. Machine learning techniques may offer more exhaustive means of cohort discovery and a novel mixed methods approach to population health.

## Appendix

Multimedia Appendix 1 Topic modelling visualization (Figure S1) illustrating the change in coherence value scores per increase in five topics (Table S1), based on EHR data of two subgroups of unplanned admissions at Chicago academic health center in 2020: 1) COVID-19 patients with unhealthy opioid use, and 2) COVID-19 patients with no unhealthy opioid use.

Multimedia Appendix 2 Topic modelling experiment to identify ten topics regarding the clinical presentations for each of two subgroups (n=4,110) of a cohort of unplanned admissions at a Chicago academic health center between January 1 and December 31, 2020 (N = 32,635): 1) admissions with COVID-19 and unhealthy opioid use (COV-UOU, n=102) and 2) admissions with COVID-19 and no unhealthy opioid use (COV-NO-UOU, n=4,008).

## Abbreviations

BERT: bidirectional encoder representations from transformers
cTAKES: Clinical Text and Knowledge Extraction System version 4.0
CUI: concept unique identifier
CV: coherence value
ED: emergency department
EHR: electronic health record
ICD-10: International Classification of Disease, Tenth Revision
ICU: intensive care unit
LDA: latent Dirichlet allocation
MOUD: medication for opioid use disorder
OR: odds ratio
OUD: opioid use disorder
RUMC: Rush University Medical Center
SMART-AI: Substance Misuse and Referral to Treatment Artificial Intelligence
SUD: substance use disorders
UAU: unhealthy alcohol use
UOU: unhealthy opioid use
