# Supplementary material for: Machine Learning Techniques to Explore Clinical Presentations of COVID-19 Severity and to Test the Association With Unhealthy Opioid Use: Retrospective Cross-sectional Cohort Study
Source: JMIR Public Health Surveill. 2022 Dec 8;8(12):e38158. doi: 10.2196/38158 (PMC9746674; doi:10.2196/38158)
Supplement: Multimedia Appendix 1 [file publichealth_v8i12e38158_app1.docx]

**Figure S1.** Topic modelling visualization illustrating the change in coherence value scores per increase in five topics, based on EHR data of two subgroups of unplanned admissions at Chicago academic health center in 2020: 1) COVID-19 patients with unhealthy opioid use and 2) COVID-19 patients with no unhealthy opioid use.


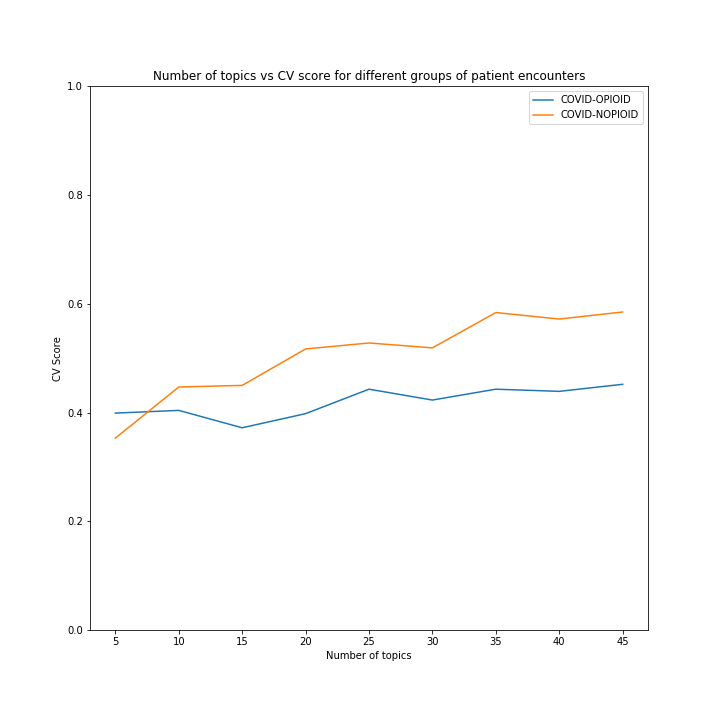


**Table S1.** Topic modelling coherence value scores per increase in five topics, based on clinical EHR data of two subgroups of unplanned admissions at Chicago academic health center in 2020: 1) COVID-19 patients with unhealthy opioid use (UOU) and 2) COVID-19 patients with no UOU.

| Number of Topics | COVID-19 UOU | COVID-19 No UOU |
| --- | --- | --- |
| 5 | .399 | .353 |
| 10 | .404 | .447 |
| 15 | .372 | .450 |
| 20 | .398 | .517 |
| 25 | .443 | .528 |
| 30 | .423 | .519 |
| 35 | .443 | .584 |
| 40 | .439 | .572 |
| 45 | .452 | .585 |
