# Supplementary material for: Machine Learning Techniques to Explore Clinical Presentations of COVID-19 Severity and to Test the Association With Unhealthy Opioid Use: Retrospective Cross-sectional Cohort Study
Source: JMIR Public Health Surveill. 2022 Dec 8;8(12):e38158. doi: 10.2196/38158 (PMC9746674; doi:10.2196/38158)
Supplement: Multimedia Appendix 2 [file publichealth_v8i12e38158_app2.pptx]

## Slide 1
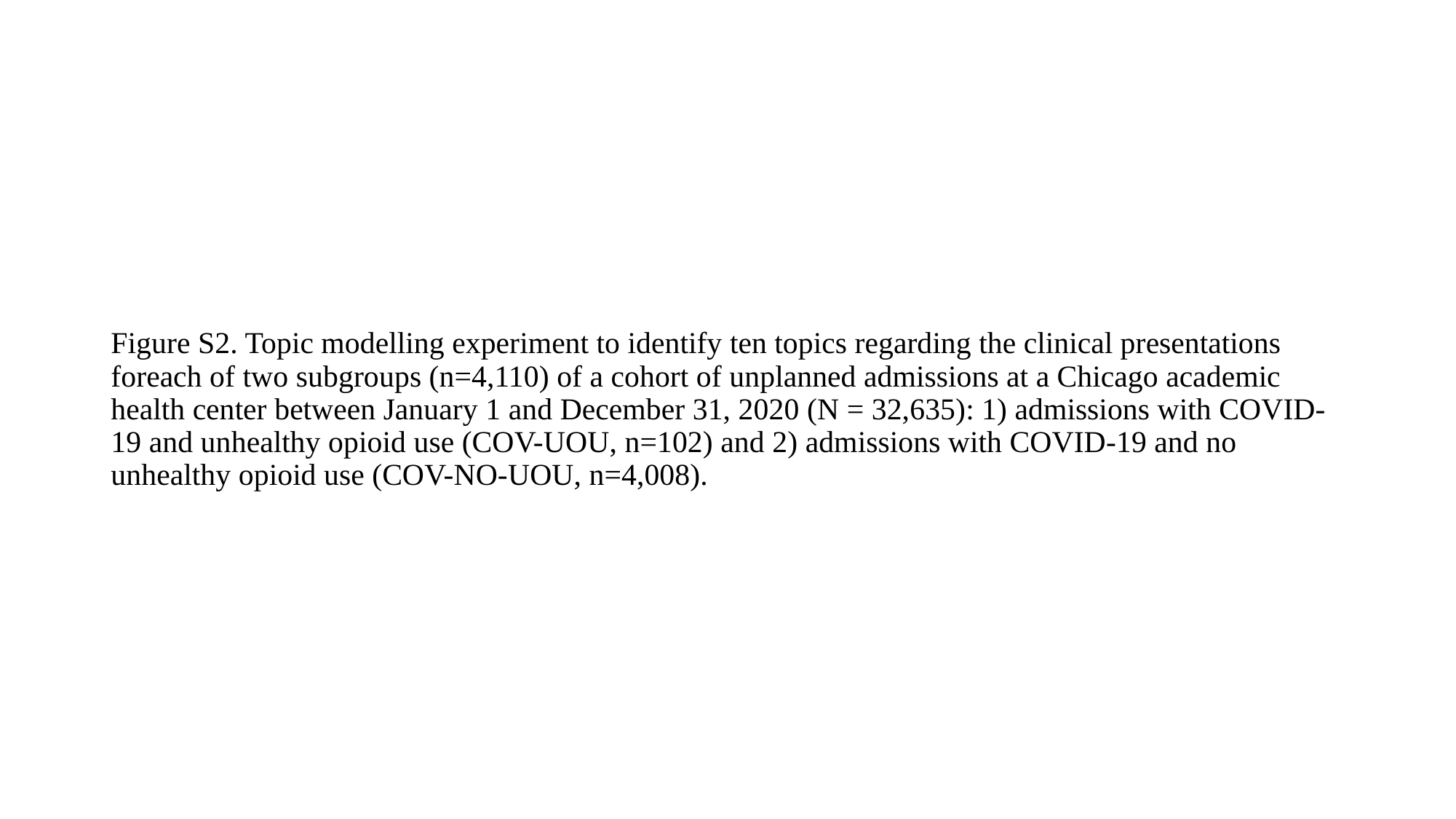

# Figure S2. Topic modelling experiment to identify ten topics regarding the clinical presentations foreach of two subgroups (n=4,110) of a cohort of unplanned admissions at a Chicago academic health center between January 1 and December 31, 2020 (N = 32,635): 1) admissions with COVID-19 and unhealthy opioid use (COV-UOU, n=102) and 2) admissions with COVID-19 and no unhealthy opioid use (COV-NO-UOU, n=4,008).

## Slide 2
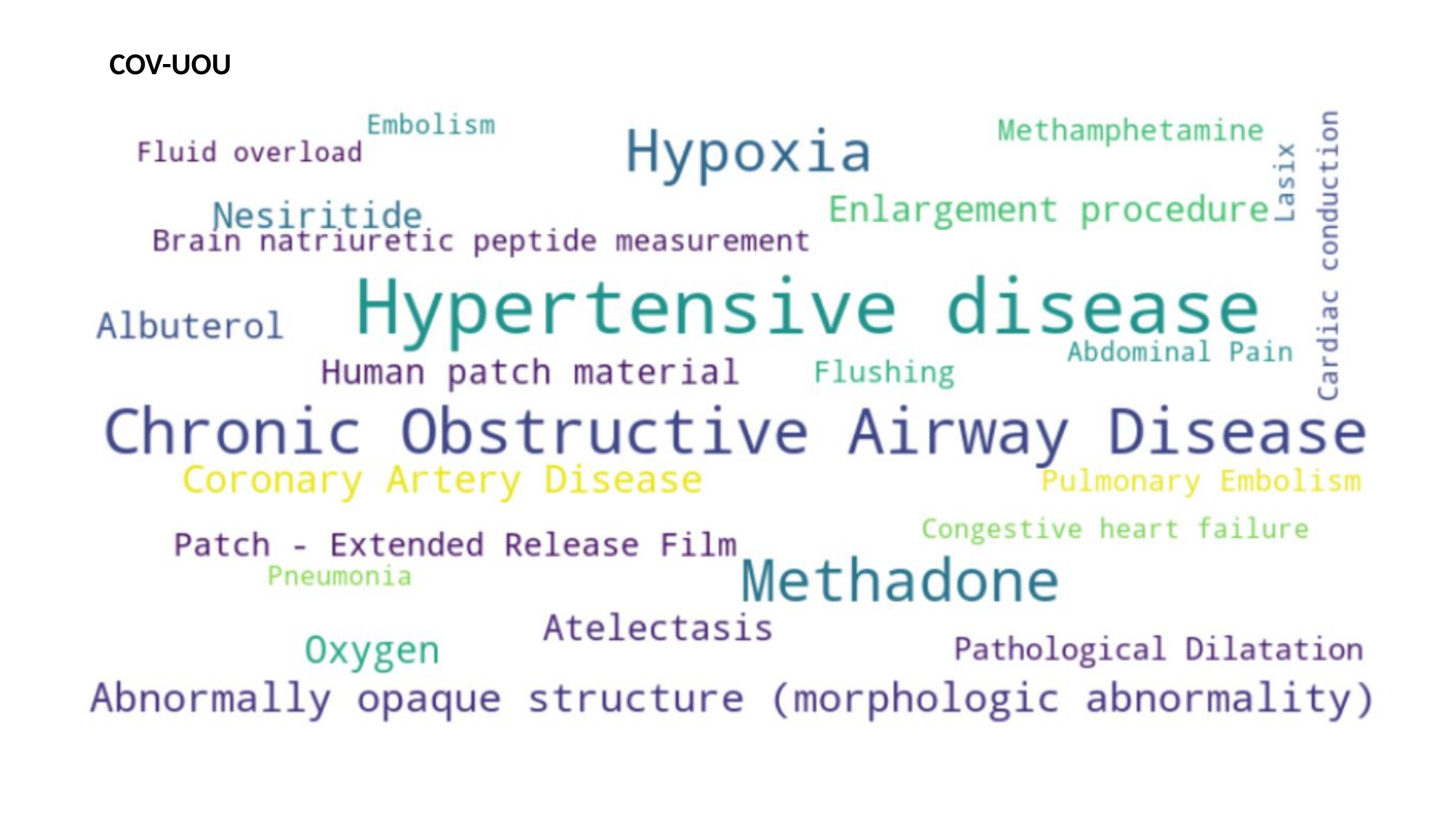

COV-UOU

## Slide 3
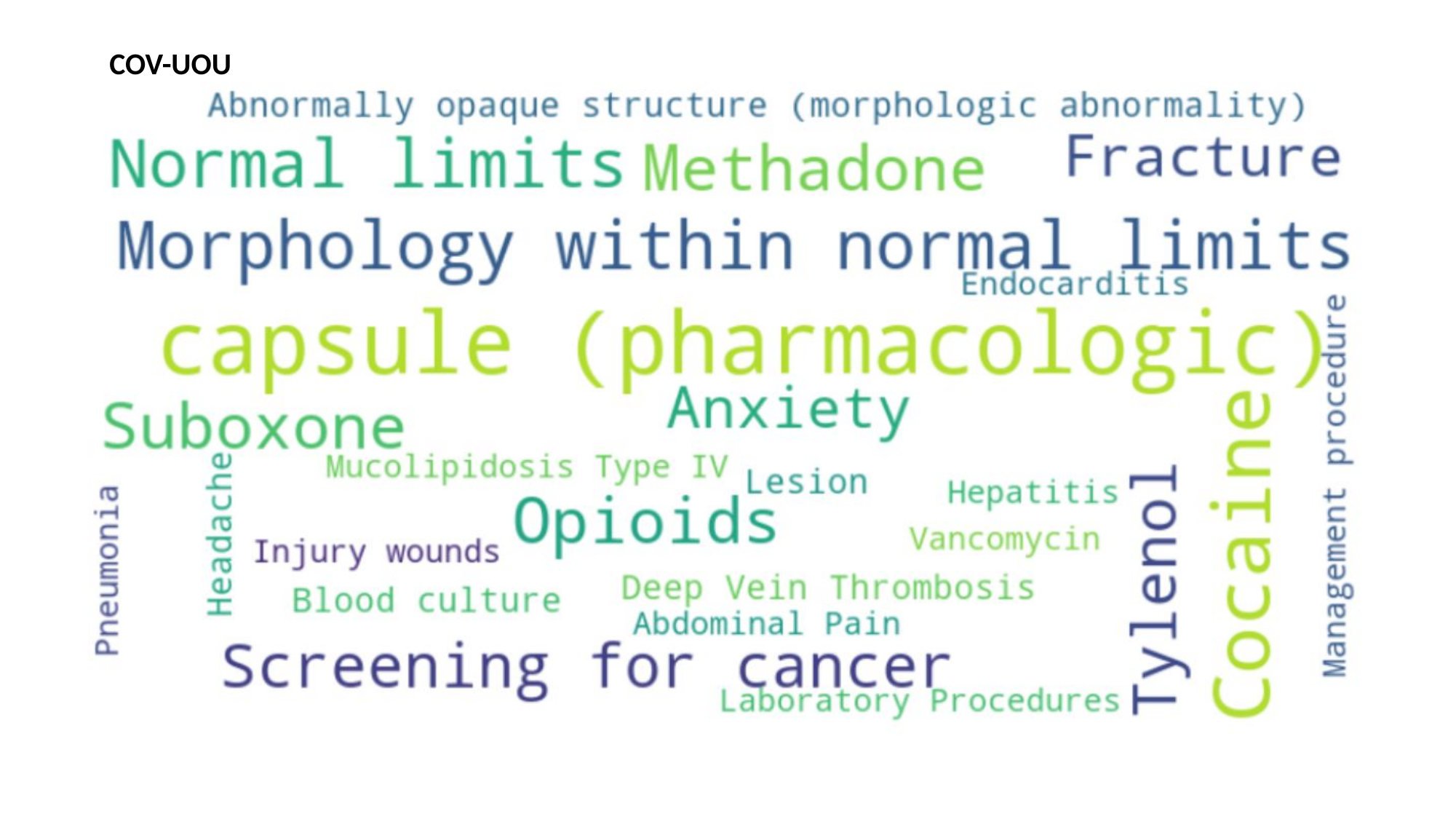

COV-UOU

## Slide 4
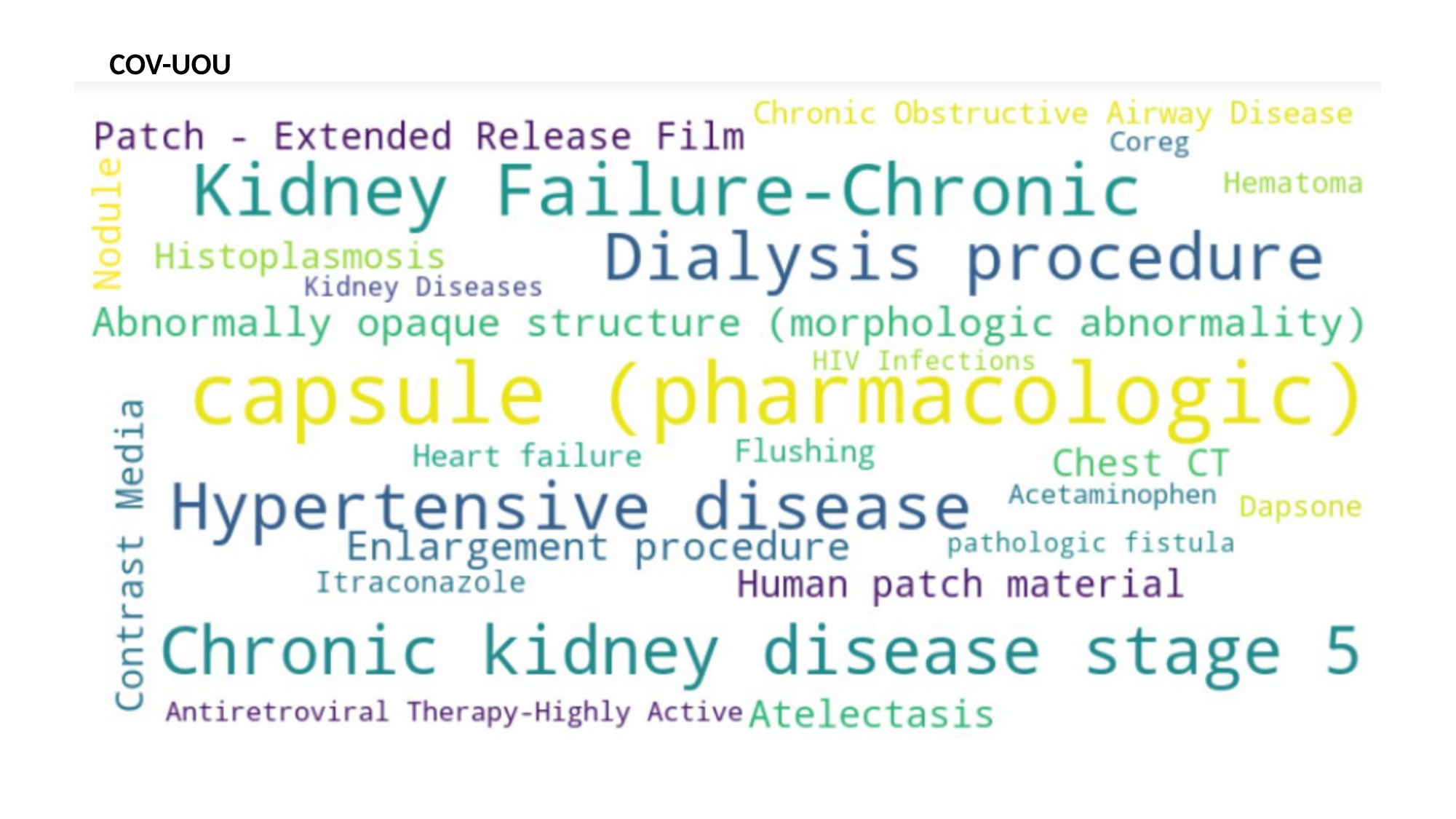

COV-UOU

## Slide 5
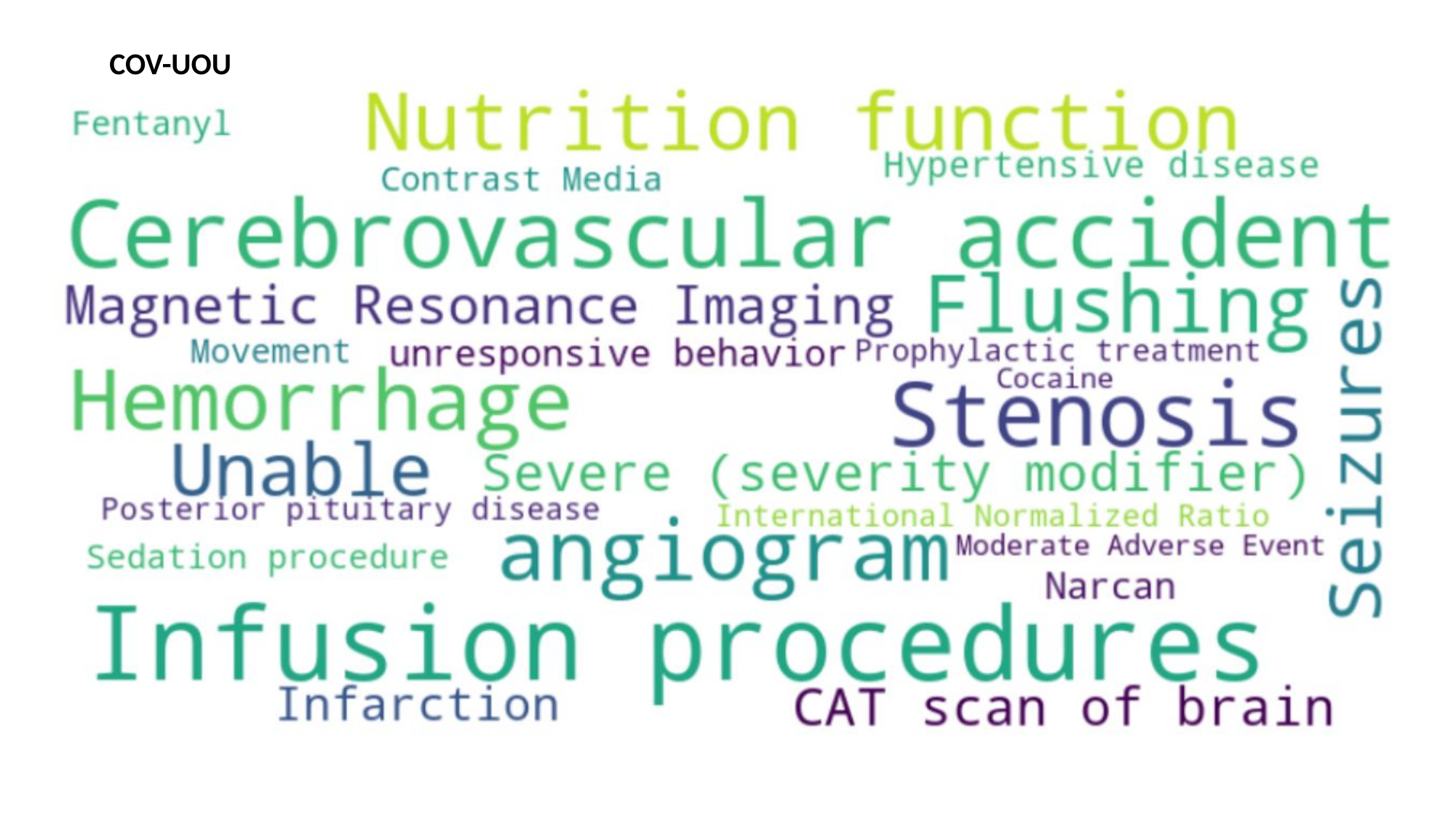

COV-UOU

## Slide 6
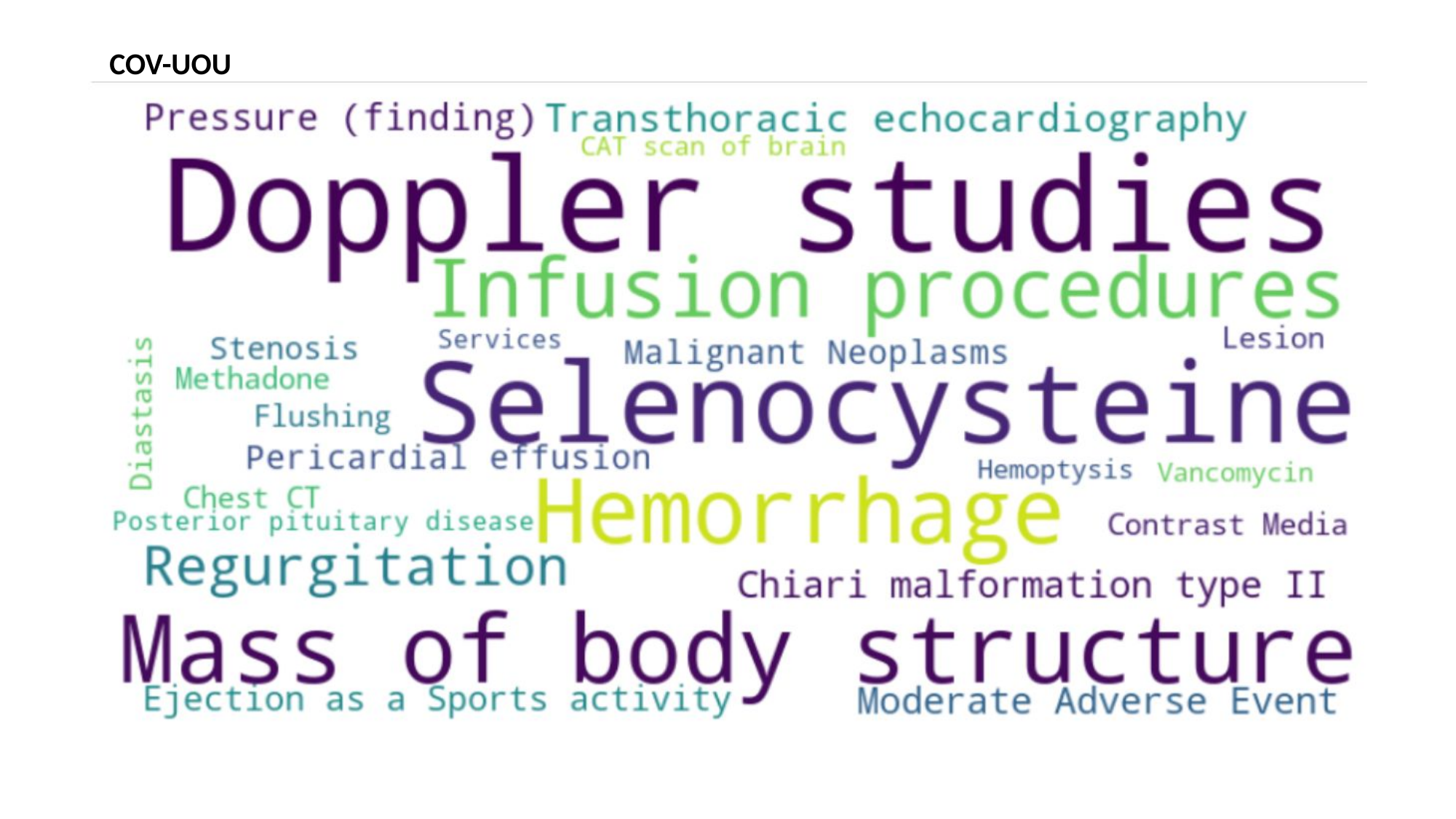

COV-UOU

## Slide 7
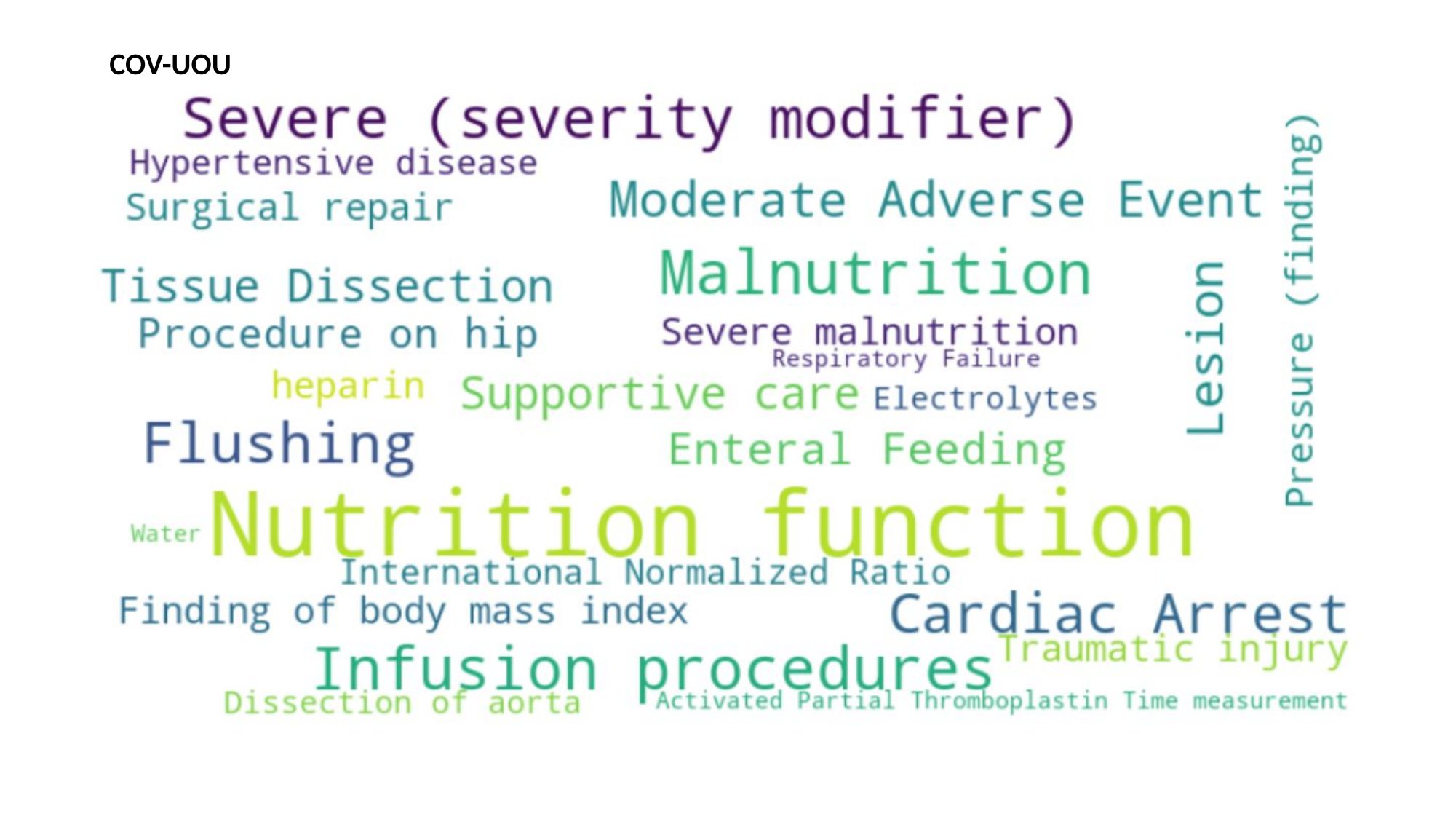

COV-UOU

## Slide 8
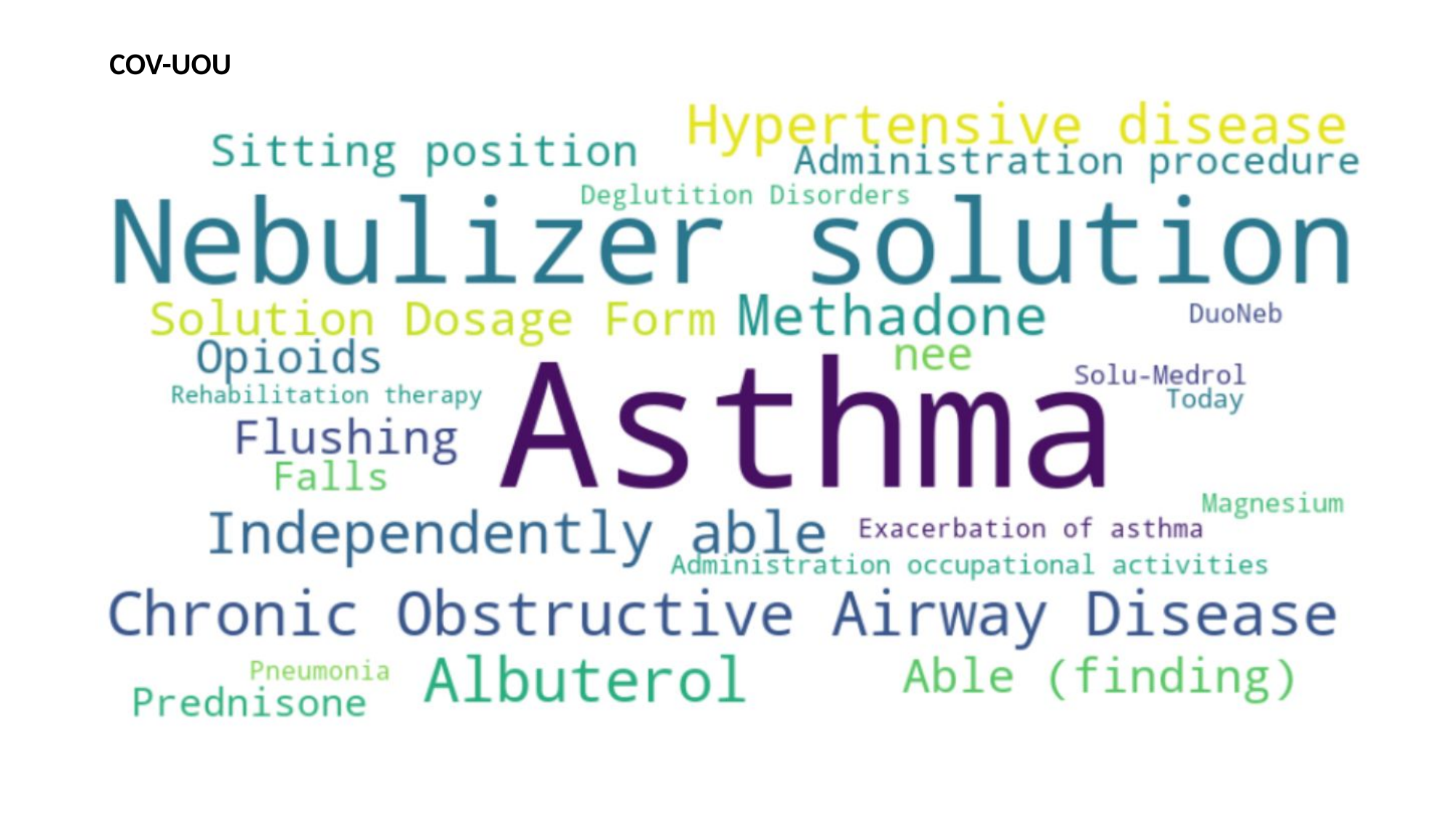

COV-UOU

## Slide 9
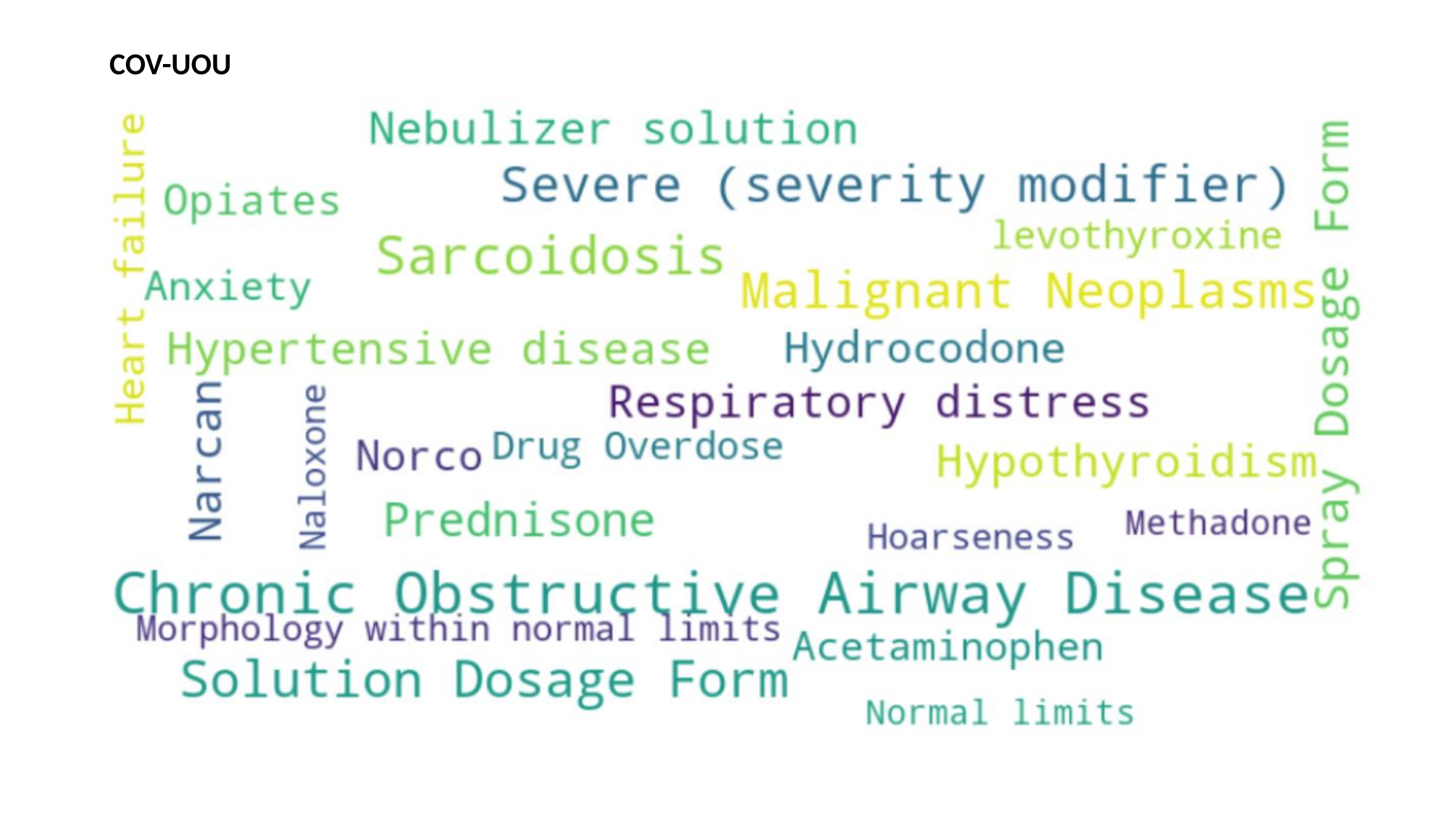

COV-UOU

## Slide 10
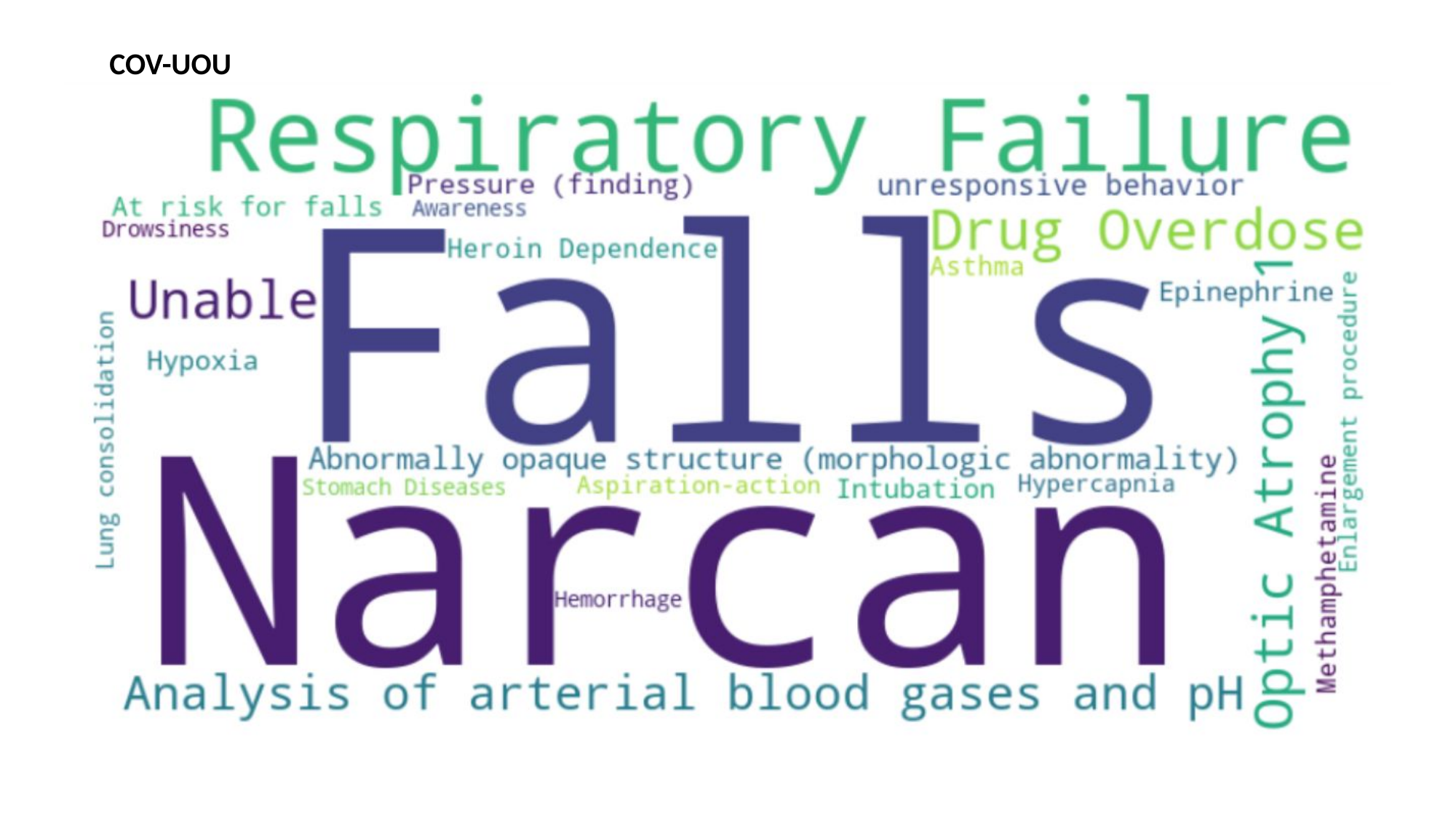

COV-UOU

## Slide 11
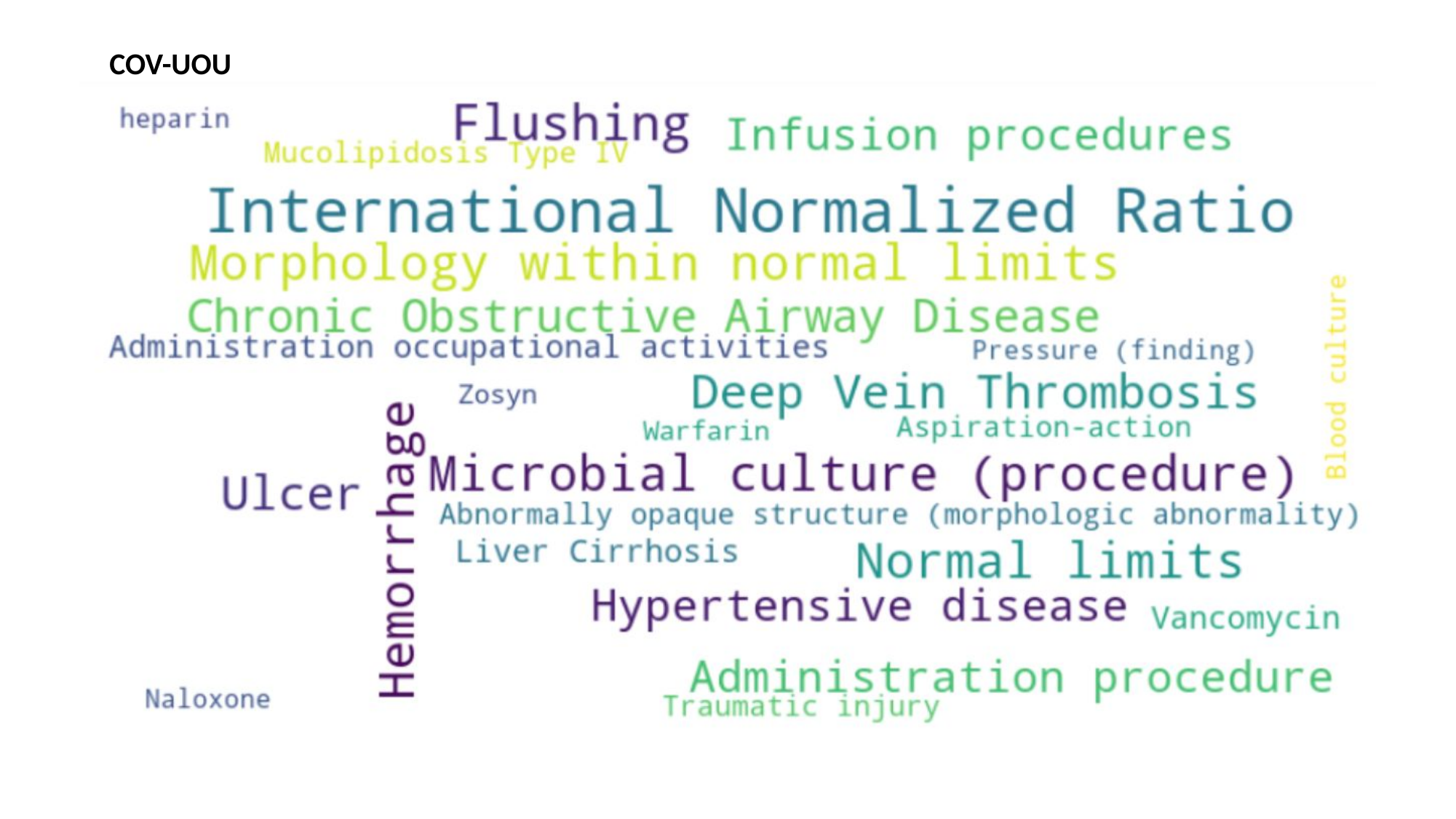

COV-UOU

## Slide 12
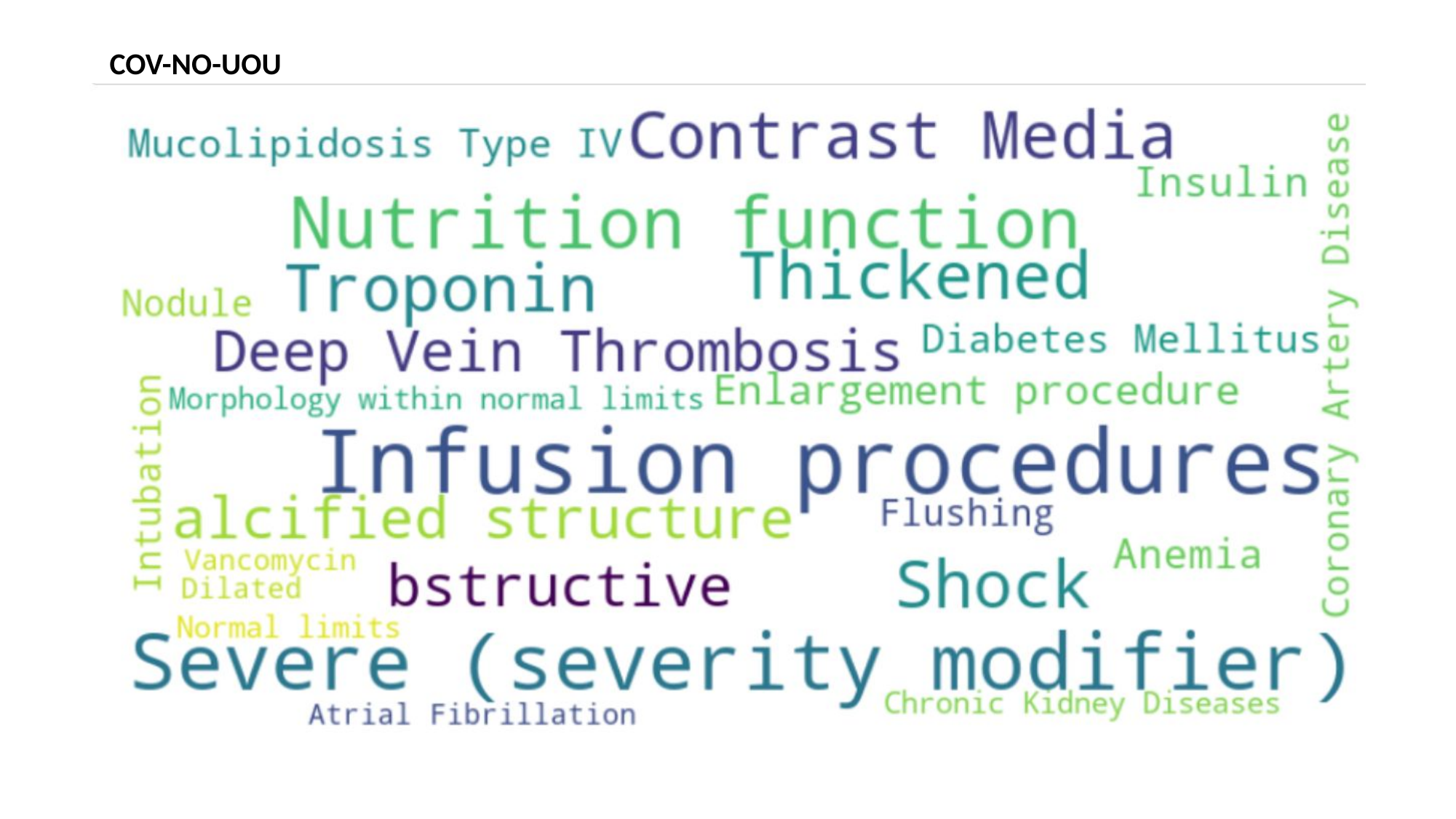

COV-NO-UOU

## Slide 13
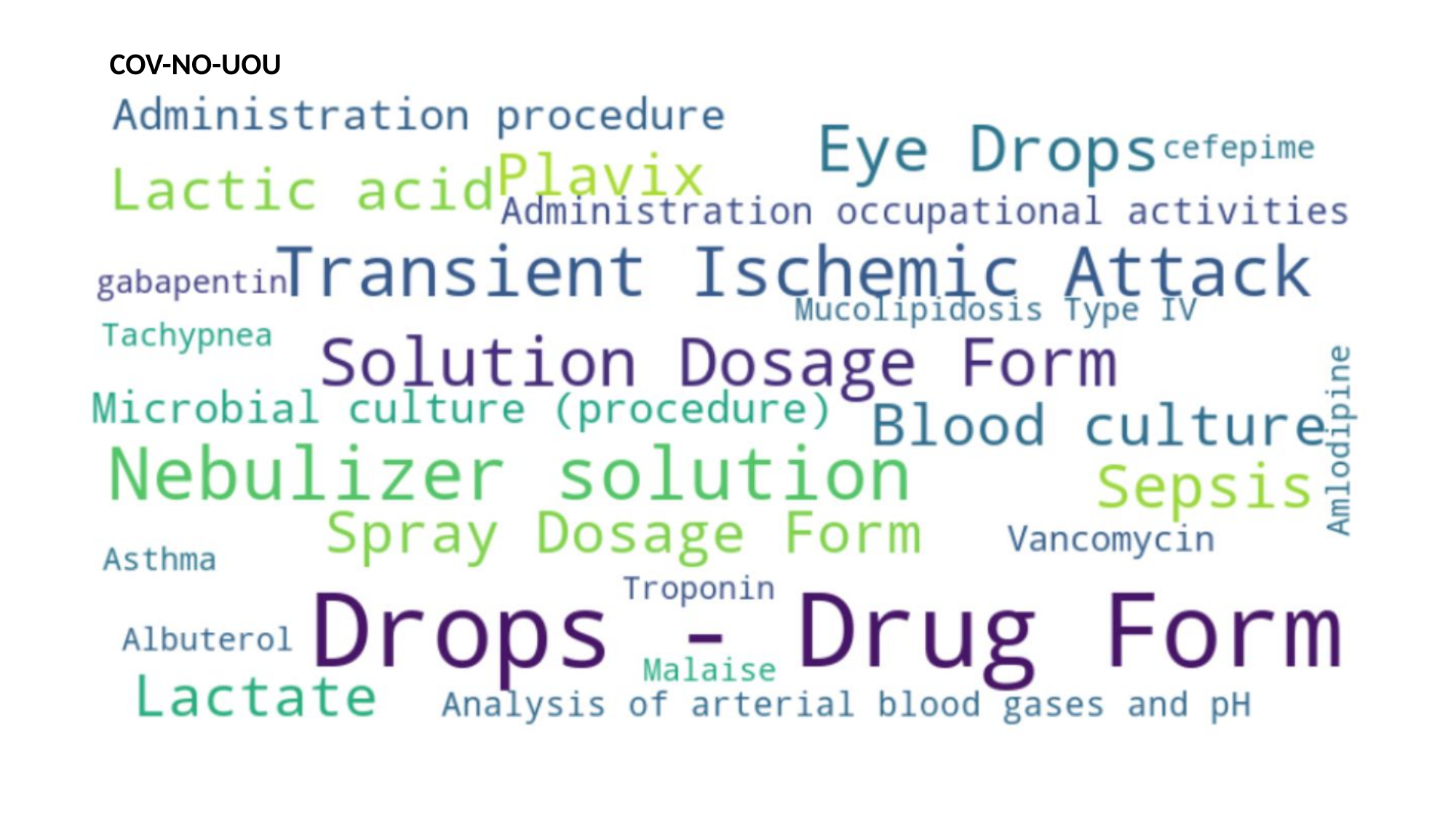

COV-NO-UOU

## Slide 14
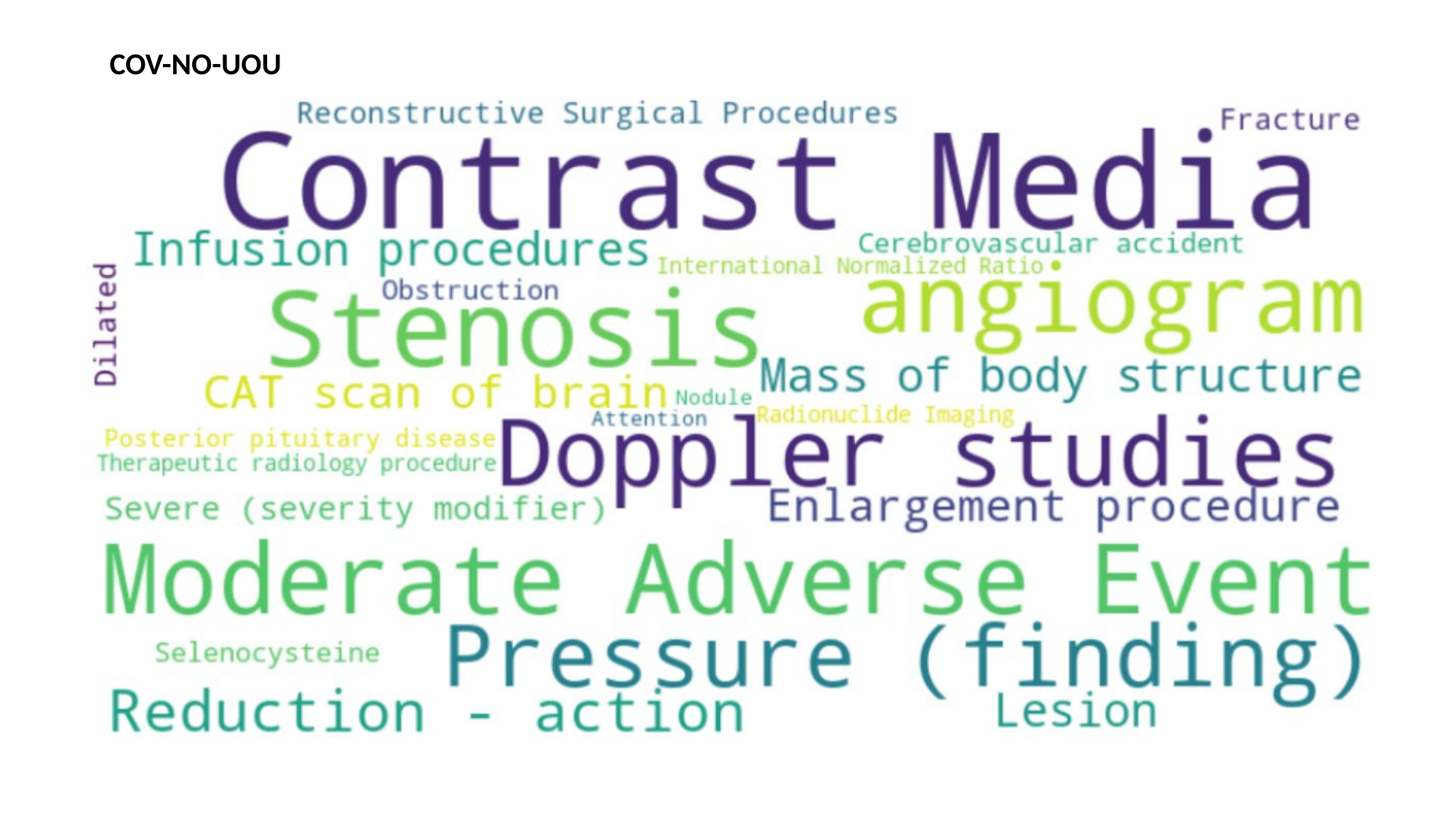

COV-NO-UOU

## Slide 15
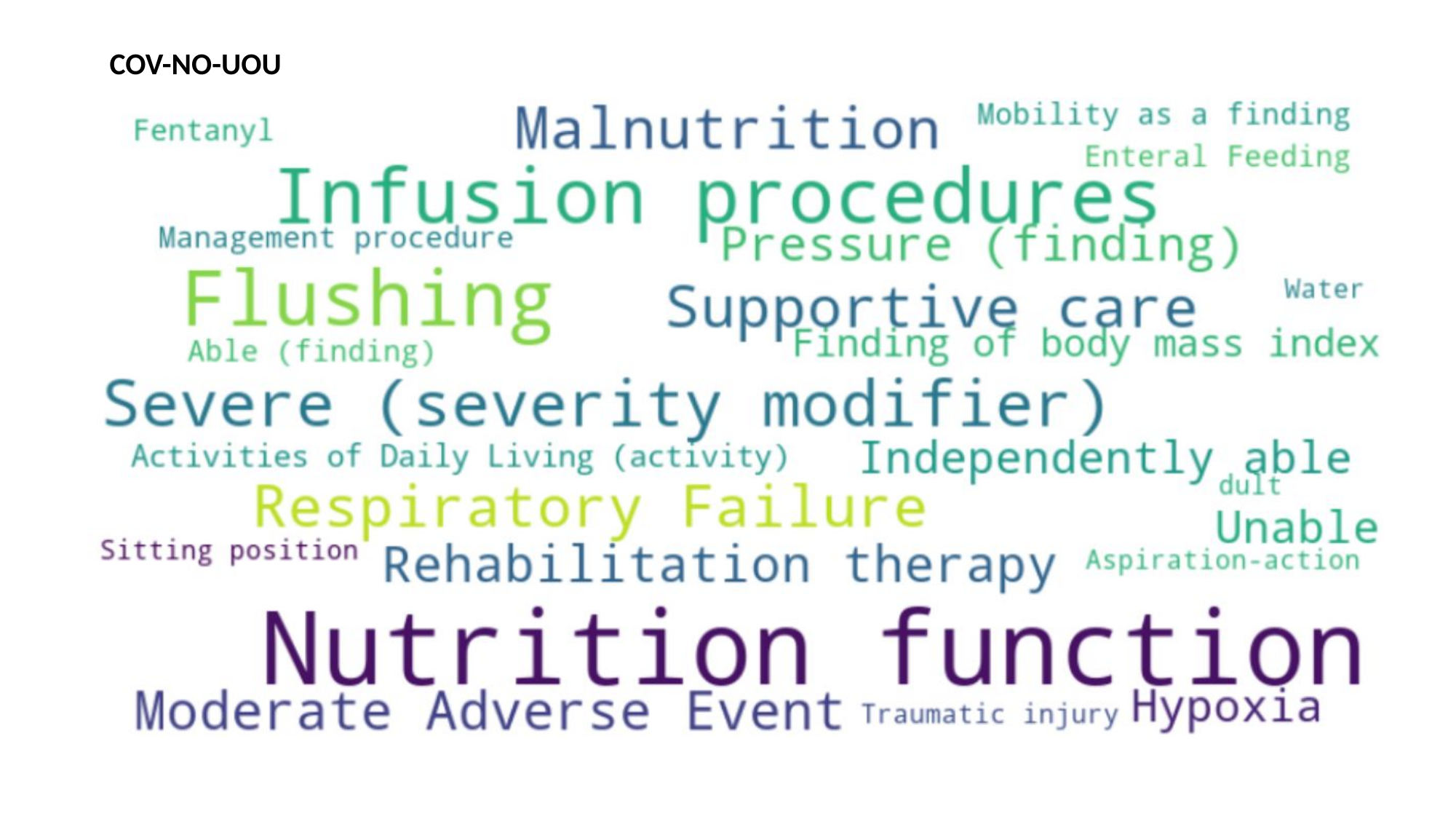

COV-NO-UOU

## Slide 16
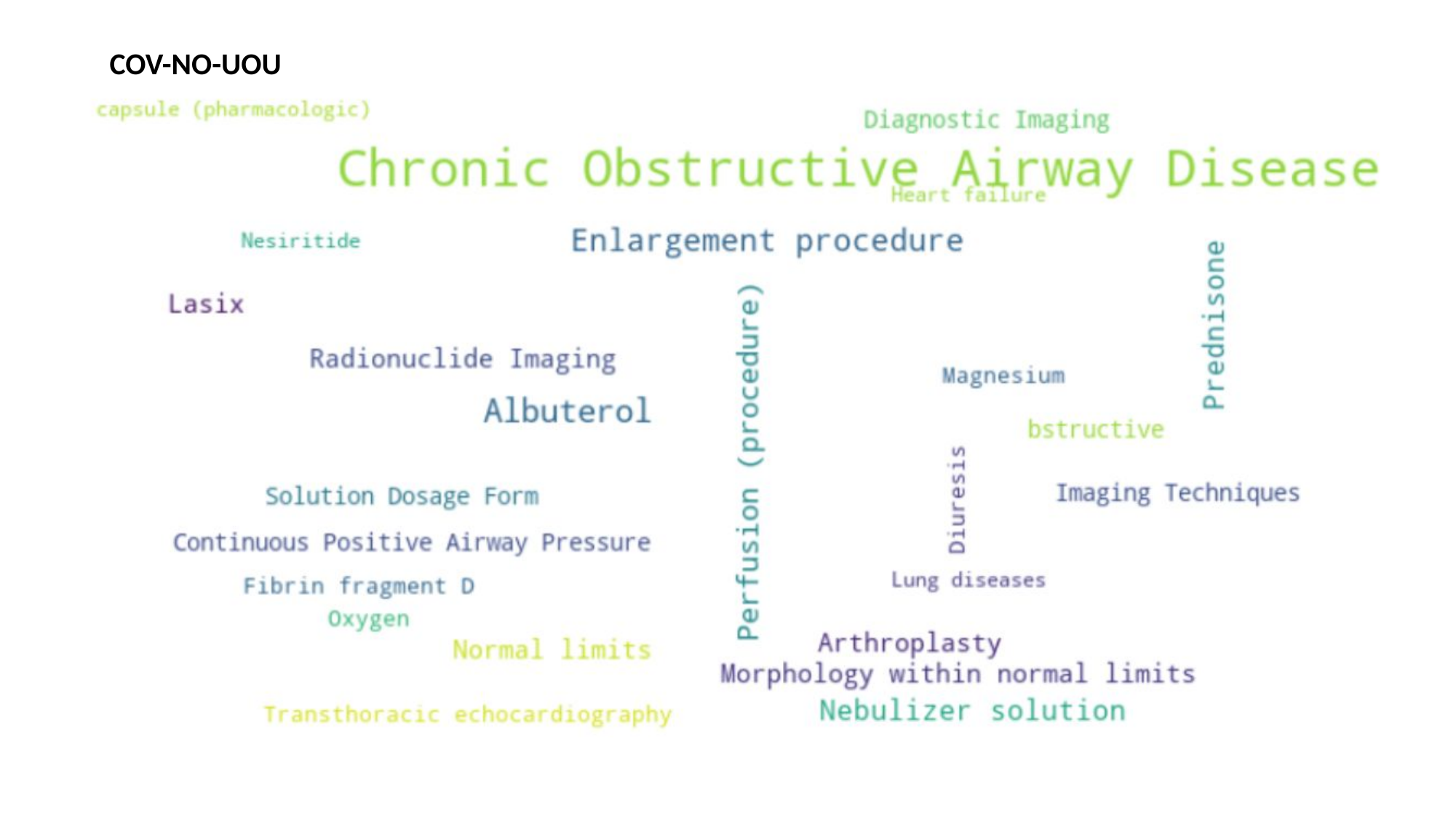

COV-NO-UOU

## Slide 17
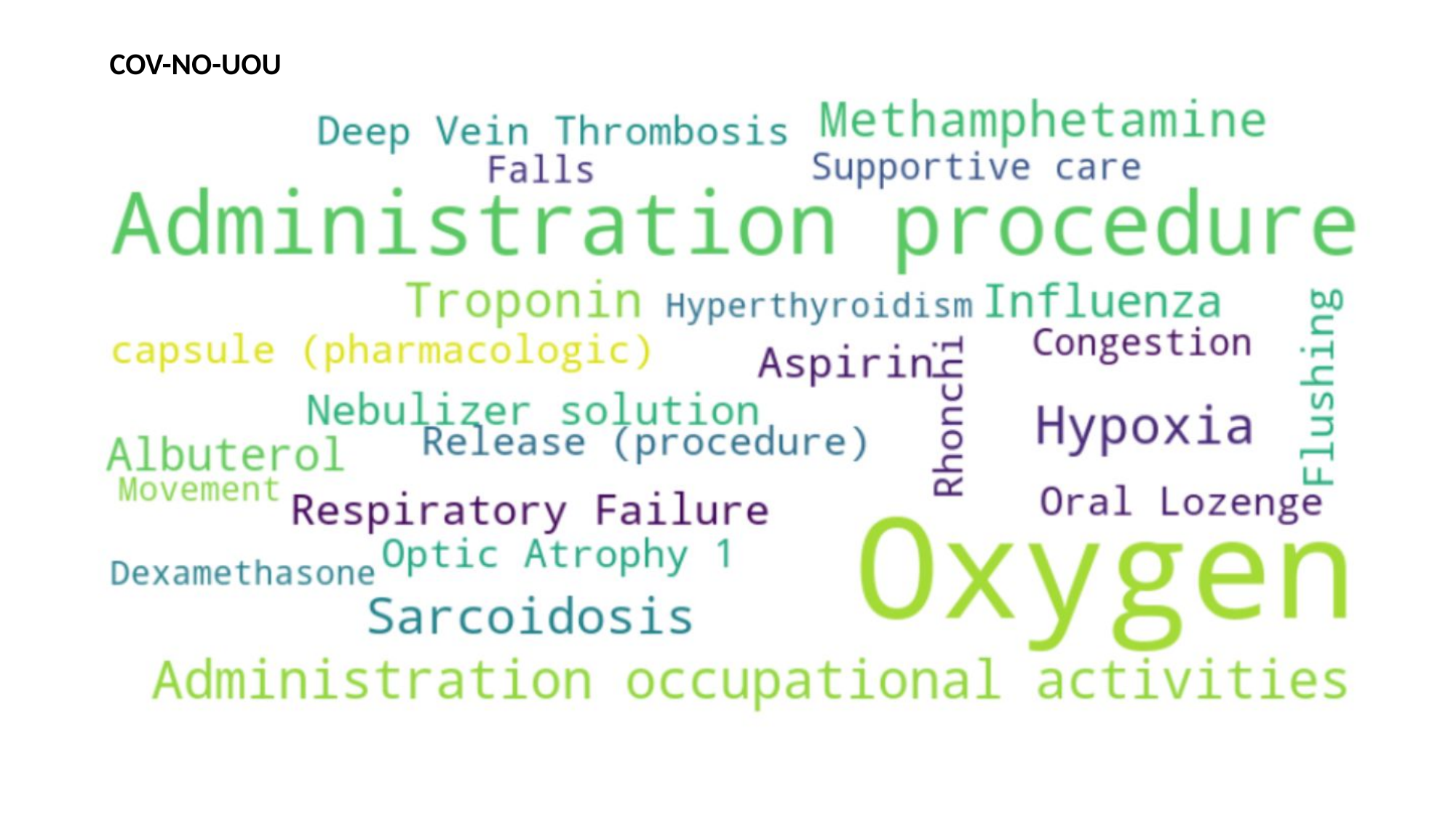

COV-NO-UOU

## Slide 18
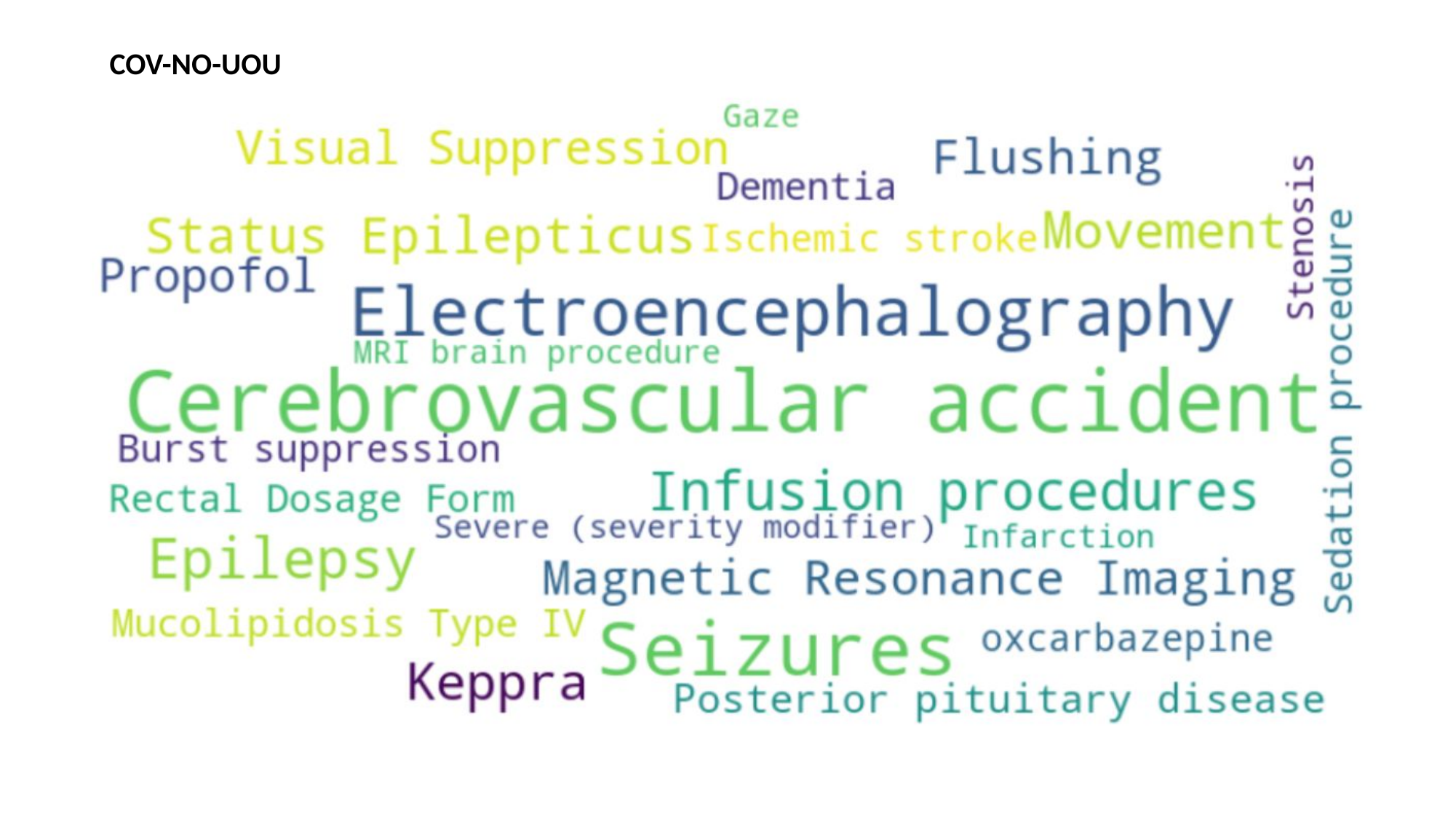

COV-NO-UOU

## Slide 19
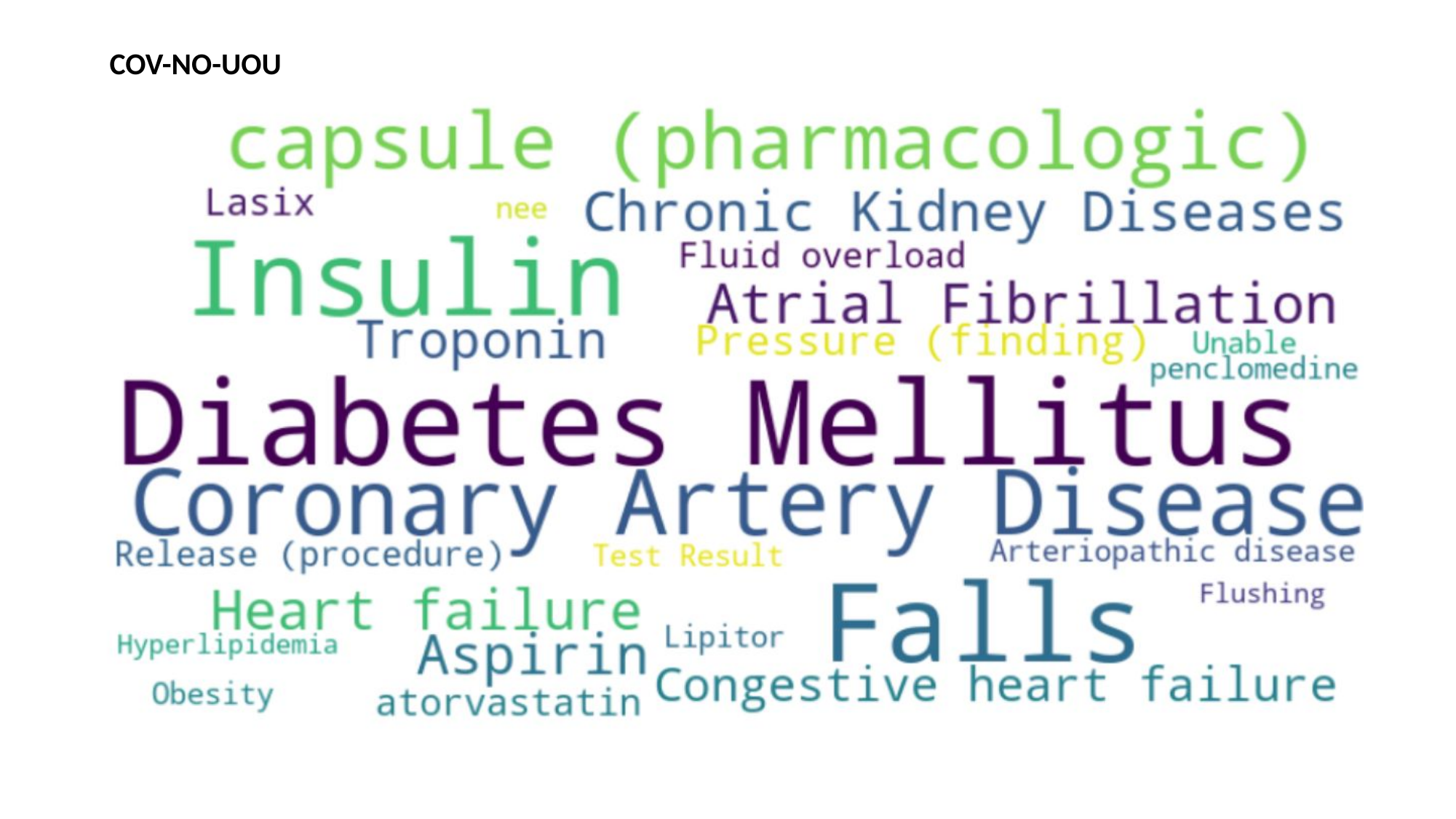

COV-NO-UOU

## Slide 20
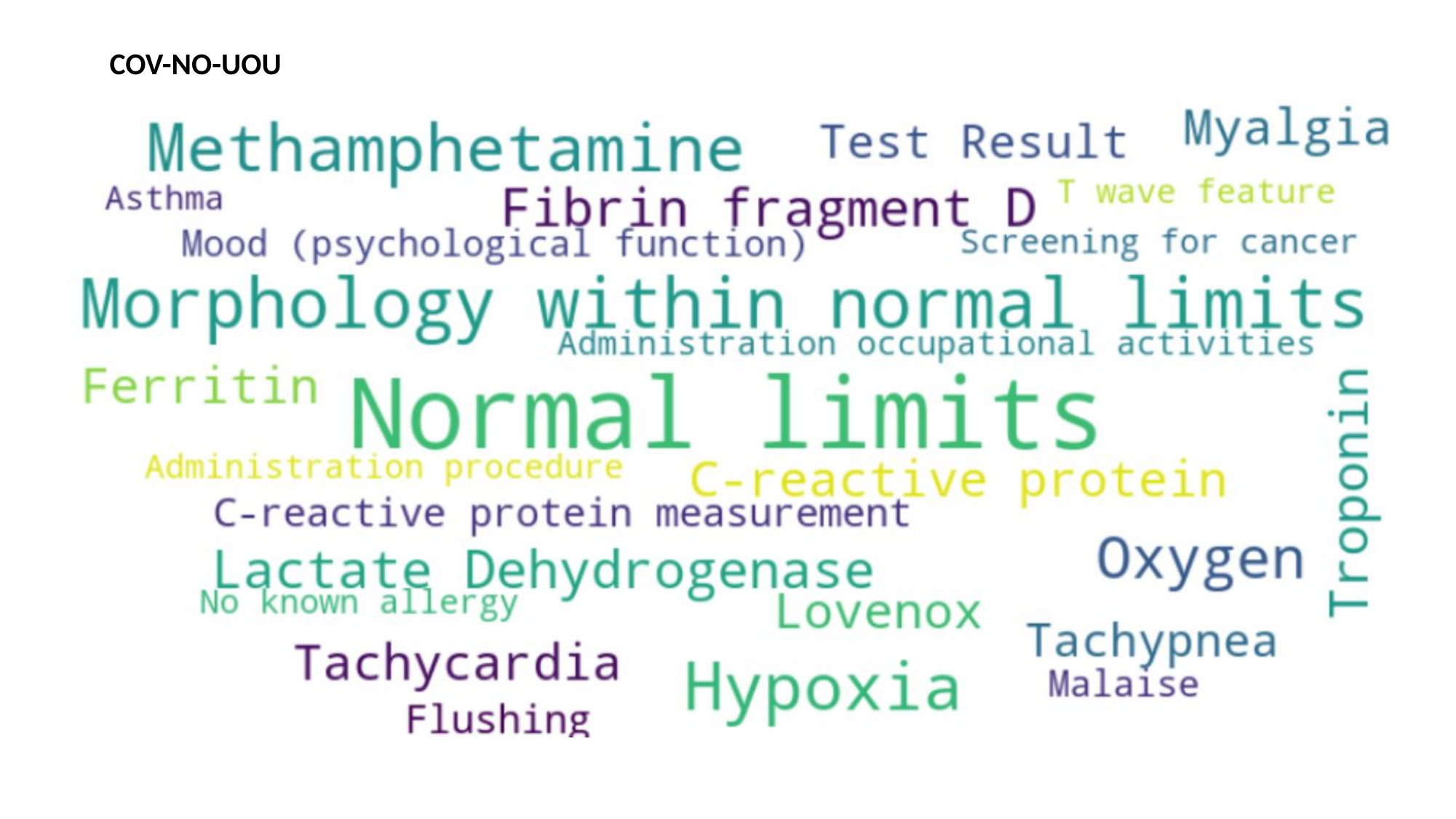

COV-NO-UOU

## Slide 21
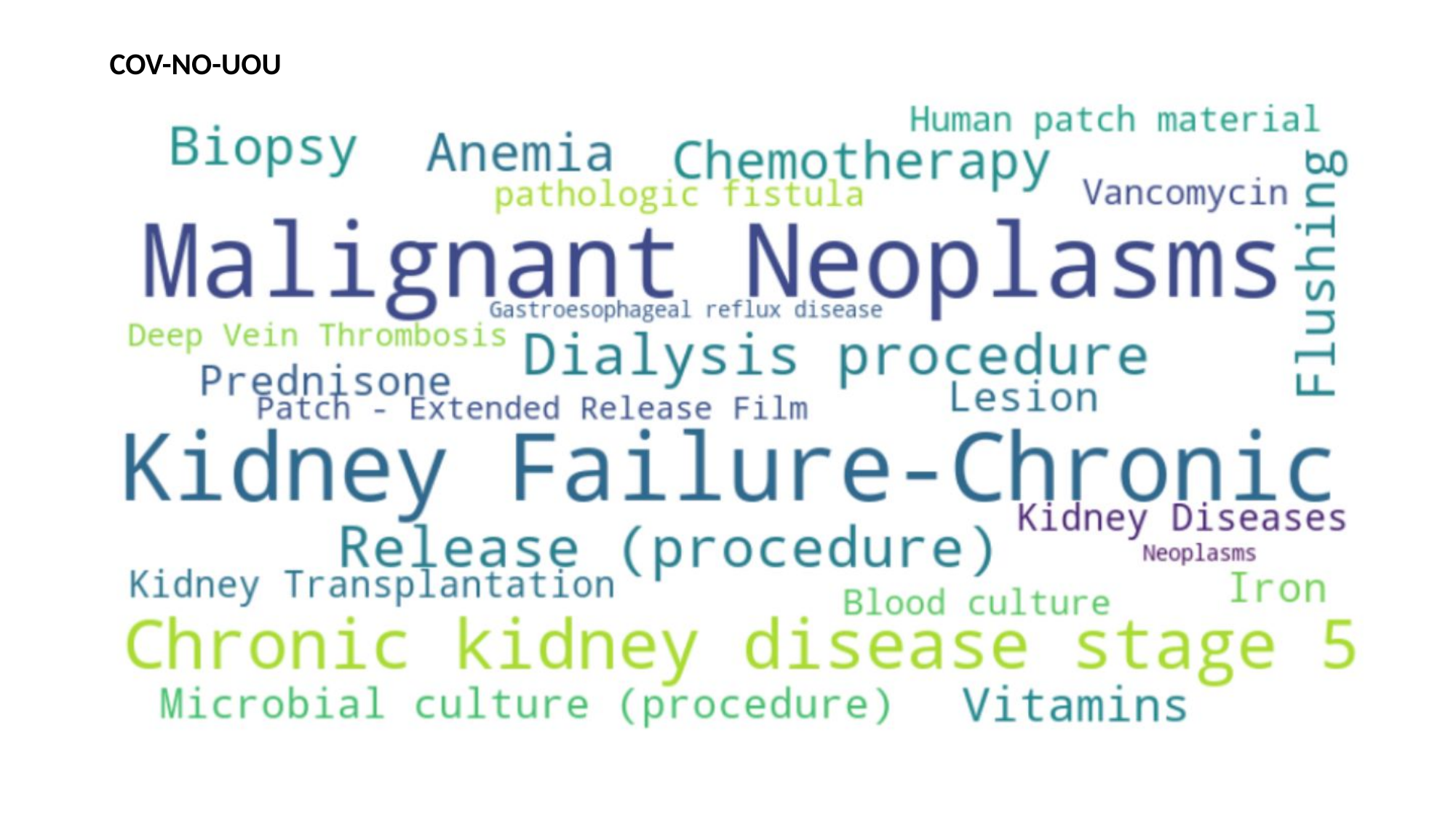

COV-NO-UOU
